# Augmenting large language models with psychologically grounded models of causal reasoning for planning under uncertainty

**DOI:** 10.3389/frai.2025.1730614

**Published:** 2026-01-30

**Authors:** Semanti Basu, Moon Hwan Kim, Semir Tatlidil, Tom Williams, Steven Sloman, Ruth Iris Bahar

**Affiliations:** 1 Computer Science, Brown University, Providence, RI, United States; 2 Cognitive and Psychological Sciences, Brown University, Providence, RI, United States; 3 Computer Science, Colorado School of Mines, Golden, CO, United States

**Keywords:** human causal models, LLM, planning under uncertainty, POMDP, robot planning

## Abstract

Large Language Models (LLMs) have come a long way in their ability to solve a wide range of problems. Yet, LLM decision-making still relies primarily on pattern recognition, which may limit its ability to make sound decisions under uncertainty. In contrast, human reasoning often makes use of explicit causal models, allowing humans to explain, hypothesize, and extrapolate to different domains in uncertain scenarios. In this article, we explore whether human causal models can be strategically integrated with Large Language Models to improve planning outcomes under uncertainty for object assembly and troubleshooting tasks modeled as Partially Observable Markov Decision Processes (POMDPs). Our contributions consist of two parts: (1) an interactive LLM agent that plans an action at each time step by solving a POMDP targeted at an object assembly or troubleshooting task, and (2) a novel hybrid-reasoning framework that uses confidence scores in both the LLM agent's output and a human causal model to make a final decision on the most appropriate action for the current time step to achieve the task. We demonstrate the efficacy of our approach through detailed simulations and show a significant improvement in task planning reward across three different state-of-the-art LLMs when augmenting the baseline LLM planner with a human causal model.

## 1 Introduction

Large language models (LLMs) have demonstrated strong performance across several domains, reaching and even surpassing human-expert level performance on certain benchmarks. However, despite recent progress in creating more powerful LLMs, their causal reasoning abilities are still deficient (Chi et al., 2024; Wu et al., 2024; Yu et al., 2025). More recently, there has been a paradigm shift in LLM research with the advent of large reasoning models (LRMs) such as OpenAI-o1, Gemini 2.5-pro, OpenAI-o3, and Deepseek-v1, which have exhibited better performance than traditional LLMs across several complex domains. The success of these large reasoning models has been attributed to training them on high-quality human-annotated step-by-step reasoning data, process or outcome reward models trained on domain-specific data that score an LLM's intermediate steps or final answer, test-time compute scaling, or a combination of these strategies. Although these LLMs perform well on coding, mathematical, and STEM benchmarks that require substantial reasoning, there remain fundamental differences between LLM reasoning and human causal reasoning.

Despite recent progress, LLM decision-making still relies primarily on pattern recognition, while human reasoning often makes use of explicit causal models (Lake et al., 2017; Sloman, 2005). Causal models allow humans to explain their observations, hypothesize about imagined scenarios through counterfactual reasoning, and draw parallels between different domains. This ability gives humans a strong foundation for decision making under uncertainty (Glymour, 2001; Krynski and Tenenbaum, 2003, 2007).

If LLMs could be augmented with human-in-the-loop causal reasoning, better decisions could be made, especially for solving problems in uncertain scenarios. To this end, we propose a reasoning framework that combines the extensive knowledge base and sophisticated pattern recognition afforded by LLMs with a human's ability to form causal mental models in order to solve sequential decision-making problems under uncertainty.

The problem-solving abilities of LLMs have been measured through a range of different benchmarks, designed to test various facets of an LLM's strengths. For example, the GPQA benchmark (Rein et al. 2023) tests reasoning and knowledge of LLMs on graduate-level science questions, SWE-bench OpenAI (2024b) assesses coding prowess, while Humanity's last exam (Phan et al., 2025) offers a multi-modal benchmark covering a variety of subject areas. These benchmarks have questions/problems with verifiable answers. In contrast, in this study, we deal with sequential decision-making problems that require optimization and planning under uncertainty. The aforementioned popular benchmarks do not measure an LLM's ability to plan under uncertainty in partially observable settings.

However, recent studies have shown that LLM-based planners perform well on different robotics tasks, including those in partially observable settings (Ahn et al., 2022; Huang et al., 2022; Sun et al., 2024; Ren et al., 2023). This leads us to hypothesize that LLMs can serve as a planner for high-level sequential decision making for assembly and troubleshooting under uncertainty. Furthermore, we propose to augment LLMs with expert causal reasoning models from humans in a way that allows for tradeoffs between an LLM's knowledge and training and a human's reasoning, and theorize that it will improve overall cumulative rewards.

In this article, we explore the ability of LLMs to act as decisi on-making agents under partial observability for solving object assembly and troubleshooting problems formulated as partially observable Markov decision processes (POMDPs). We further investigate whether human mental causal models of objects integrated with LLMs can improve overall performance. In the context of object assembly and troubleshooting, improved performance is interpreted as fully assembling or repairing the object in as few sequential steps as possible. To that end, in this study, we seek to answer the following research questions:

- **RQ1: Can LLM agents solve POMDPs effectively?** We designed an interactive LLM agent that plans an action by solving a POMDP targeted at either an object assembly or a troubleshooting task. We evaluated the agent using state-of-the-art large language “reasoning” models (o3-mini, o4-mini) as well as “non-reasoning” models (GPT-4o).
- **RQ2: Can LLM agents be integrated with human expert causal models for trust-based conflict resolution for improved decision making?** To investigate this research question, we explore whether rewards obtained by solving the POMDP can be improved by creating a decision-making framework that weighs an LLM's decision against a human's causal model to make the final call on the action planned at each time step.

We note that with regards to the second research question, the focus of this study is not on the construction of the causal model itself, but on its use for better decision making with LLM agents. As discussed later in Section 3.3, the human causal models used in this study are constructed using the approach described in our prior works (Basu, 2025; Tatlidil et al., 2025).

## 2 Related work

### 2.1 LLM agents as planners

There is a growing body of studies on using LLMs as planners. In SayCan (Ahn et al., 2022), an LLM is used to generate a high-level plan, while a learned value function determines if it is feasible to execute the proposed action. ReAct (Yao et al., 2023b) introduces a framework that allows an LLM agent to alternate between generating reasoning traces and acting to solve tasks. Reflexion (Shinn et al., 2023) introduces a method to convert environmental signals/observations into useful feedback that is incorporated into an LLM agent's context, allowing it to learn from prior mistakes while planning. Inner Monologue (Huang et al., 2022) demonstrates how LLM agents in robot planning can leverage feedback from perception and detection modules for improved task completion. In (Zhao et al. 2023), LLMs are used to build a world model for POMDPs, which is then used by a Monte Carlo Tree Search (MCTS) based algorithm for planning. The search is further informed by using LLMs as a policy. Another framework using LLMs in partially observable robotics domains is introduced in (Sun et al. 2024), which proposes using the LLM as the policy model as well as for state abstraction. KnowNo (Ren et al., 2023) is a framework that proposes using conformal prediction for uncertainty alignment in LLM-based planners in partially observable tasks.

Our work is similar to these approaches mentioned above in the sense that we also propose to use LLMs as policy in an embodied task, similar to (Ahn et al. 2022); (Huang et al. 2022). Our task is also in a partially observable setting, similar to (Zhao et al. 2023); (Sun et al. 2024); (Ren et al. 2023). However, in contrast to prior studies, we propose using human causal models to serve the dual purpose of (1) grounding the LLM in the physical world, and (2) supplementing the LLM with causal reasoning abilities, which it lacks.

### 2.2 Reasoning in LLMs

Several prompting strategies, such as Chain of Thoughts (Wei et al., 2022) and Tree of Thoughts (Yao et al., 2023a) have been proposed to elicit step-by-step explanations from LLMs before outputting the final answer. These strategies, in turn, have been shown to improve task performance over simple prompting. Prior work has also leveraged chains of thoughts/reasoning traces together with reward models to improve output. Training pipelines often incorporate the use of trained reward models that can signal to the LLM whether a reasoning trace results in a correct answer or whether intermediate steps generated are correct or not. These reward models can provide feedback on the final answer (outcome supervision) or on intermediate steps (process supervision). REST-MCTS* (Zhang et al., 2024) proposes a way to evaluate intermediate steps in process supervision without human labels by using a Monte Carlo tree search-based approach to infer the probability that a step eventually leads to the correct answer. The authors of (Lightman et al., 2024) argue that using process supervision produces better reward models compared to outcome supervision in mathematical reasoning tasks.

More recently, large reasoning models (LRMs) such as the OpenAI-o1 series (OpenAI, 2024c) and Deepseek-R1 (Guo et al., 2025) have been in the spotlight for dramatically improved performance over traditional LLMs in reasoning-heavy benchmarks. They build upon previous prompting techniques where asking the LLM to break down their answer in a step-by-step manner was observed to improve accuracy of the final answer. These models improve upon traditional LLM models by increasing train-time compute or by increasing test-time compute or both.

Increasing train-time compute requires training LLMs on reasoning tasks on top of the training that LLMs usually undergo. The term “reasoning trace” is commonly used in the LLM community to refer to a sequence of intermediate tokens that the LLM autoregressively generates prior to the actual answer. This series of tokens is referred to as a “chain of thought” or a “reasoning trace”, an anthropomorphization that researchers argue can be harmful and misleading (Kambhampati et al., 2025). We use “reasoning trace” to refer to the series of tokens sequentially generated by LLMs prior to the answer but do not claim that such traces reflect actual human-like reasoning. Reinforcement learning has been used to train Deepseek-R1 (Guo et al., 2025) through a verification-based reward model, which allowed the LLM to identify reasoning traces that led to a final correct answer without human annotation.

Inference time scaling allows models to deliberate longer at inference time by allocating more test-time compute resources. Parallel scaling involves sampling several reasoning traces in parallel, and choosing the best one using majority voting, best of *N* sampling, or other aggregation techniques. If a domain-specific pre-trained process reward model is available, it can be used to score intermediate steps, followed by strategic search (beam search, lookahead search or Monte Carlo tree search) to find the best answer (Snell et al., 2025). Sequential scaling involves teaching the LLM to iteratively refine its own answers by changing its internal distribution using its previous answer before sampling the next answer. Hybrid techniques combine both strategically. More details on inference time scaling here (Zhang et al., 2025).

While large reasoning models show improved performance on certain causal reasoning benchmarks, they still fall short of human performance on many other tasks (Yu et al., 2025). In (Chi et al. 2024), the authors argue that LLMs engage in shallow causal reasoning, which can be attributed to associations learned in training, but fall short on more complex scenarios. The ability of LLMs to generalize across tasks was tested in (Wu et al. 2024) through the design of counterfactual task variants. The degradation in performance of LLMs on counterfactual variants suggests the strong reliance of current models on learned data, further highlighting the lack of causal reasoning abilities in LLMs. While (Chi et al. 2024); (Wu et al. 2024) do not evaluate LRMs, they shed light on some of the limitations of the foundational architecture of LLMs (and LRMs) that prevent them from engaging in true causal reasoning and instead being reliant on training that leads to lead to high performance.

Our framework proposes to bridge this causal reasoning gap by using human-generated causal models to supplement LLMs. Many cognitive scientists believe that causal models serve as the infrastructure for human thought (Waldmann, 2017). Humans build explanatory causal models of the world (Sloman, 2005) and use these models to reason in hypothetical space, allowing for intentional causal reasoning for problem solving. LLMs (including LRMs) use trained verifiers to signal which line of reasoning is likely to produce the correct answer; hence, any causal reasoning observed is incidental. We demonstrate that if mental causal models of objects from people are combined with task-specific world knowledge of LLMs, it can improve cumulated rewards in different object-related tasks.

## 3 Methodology

In this section, we first describe how object assembly and troubleshooting tasks can be formulated as POMDPs. We then describe our prompting structure to elicit actions from LLMs to solve the aforementioned POMDPs. Finally, we describe our hybrid-reasoning framework, where we maintain a separate external belief distribution over system states embedded with human causal graphs, which allows us to (1) ground the LLM in the problem by checking for hallucinations or repetitions, and (2) systematically deal with conflicts when the LLM suggests actions that are not causally relevant according to the human's mental model.

### 3.1 Problem formulation

Object assembly is formulated as the task of sequentially connecting pairs of parts to assemble an object. The uncertainty is over part connections, i.e., it is not known *a priori* which parts should be connected to each other. The goal is to assemble the object in as few steps as possible.

Troubleshooting is defined as sequentially inspecting pairwise part connections (and the parts themselves) to identify the root cause of a malfunction. The uncertainty is defined over the true location of the malfunction.

A POMDP is defined by the tuple 〈*S, A*, Ω, *O, R, T, d*〉, where *S* represents the state space, *A* represents the actions an agent can take, Ω represents the set of observations the agent can receive from the environment, *O* is the observation model, *T* is the transition model, *R* is the reward model, and *d* is the discount factor. The full definitions of object assembly and troubleshooting can be found in (Basu et al. 2025). Our formulations for object assembly and troubleshooting as POMDPs are taken from (Basu et al. 2025). We provide a brief overview here for completeness.

#### 3.1.1 Object assembly formulated as a POMDP

##### 3.1.1.1 State space (*S*)

The state represents the state of assembly. Since assembly is a series of pairwise part connections, the state defines the pairs that were already tried and whether they could be successfully connected. The state also tracks if the assembly is in progress.

##### 3.1.1.2 Action space (*A*)

This is the set of actions that the agent can take to assemble an object. There are n2 actions for an object with *n* parts, representative of each pairwise connection that the robot can try to connect together.

##### 3.1.1.3 Observation space (Ω)

A positive observation is received if the two parts to be connected according to the action planned actually connect; otherwise, a negative observation is received.

##### 3.1.1.4 Transition model (*T*)

*T*(*s*′|*s, a*) is the probability of moving to a new state if a particular action is taken. In our case, it is deterministic. On taking an action, the state transitions to a new state where the pair of parts corresponding to the action is marked as a tried combination (and avoided in the future). We assume actions are always executed without errors. Hence, if an action of connecting two parts was successful, progress was made toward the final assembly. If not, then the current assembly structure did not change, but we obtained information on connections that are not part of the final assembly.

##### 3.1.1.5 Observation model (*O*)

*O*(*o*′|*s*′, *a*) is the probability of making an observation *o*′ if an action *a* caused the state to transition to *s*′. In our case, it is deterministic since we assume error-free action execution. We receive a positive observation if two parts can be connected, and a negative observation otherwise, with no uncertainty.

##### 3.1.1.6 Reward model (*R*(*s, a*))

A reward of −1 is awarded at each step unless an action results in a transition to a terminal state, in which case a positive reward of n2 is received. The reward function is designed so that the cumulative reward tells us the number of steps saved in assembly.

#### 3.1.2 Object troubleshooting formulated as a POMDP

##### 3.1.2.1 State space (*S*)

The state *S* represents the state of troubleshooting an object with an observable malfunction. It represents the object parts or pairwise part connections that were already inspected/replaced. It also tracks whether the object has been fixed (i.e., functioning again) or not.

##### 3.1.2.2 Action space (*A*)

The Action space consists of the set of actions the agent can take to troubleshoot the object. There are n2 “inspect” actions for an object with *n* parts, representative of each pairwise connection that could be inspected in search of the true error location. There are also *n* “replace” actions corresponding to the *n* object parts that could be potentially replaced to fix the problem.

##### 3.1.2.3 Observation space (Ω)

A positive observation is received if an action fixed the malfunction and a negative observation is received otherwise.

##### 3.1.2.4 Transition model (*T*)

On taking an action the state transitions to a new state, where the pair of parts corresponding to the action is marked as tried (and avoided in the future). Similar to assembly, we assume that actions are always executed without errors.

##### 3.1.2.5 Observation model (*O*)

In our case, *O* is deterministic as we assume error-free action execution. We receive a positive observation if an inspection/replacement fixed the malfunction and a negative observation otherwise, with no uncertainty.

##### 3.1.2.6 Reward model (*R*(*s, a*))

A reward of −1 is awarded at each step unless an action resulted in a transition to a terminal (i.e., repaired) state, in which case a positive reward of n2+n is received. The reward function is designed so the cumulative reward indicates the number of steps saved in troubleshooting.

Solving a POMDP involves mapping a belief to an action at each time step. The belief (*B*) is necessary to capture the uncertainty over states introduced through partial observability. It captures the probability of a state being the “true state” given the history of actions and observations (*h*) taken up to that time step (i.e., *B*(*h*) = *Pr*(*s*_*t*_ = *s*_*true*_|*h*)). The agent executes the action, which produces an observation from the environment. The action and observation together are used to perform a Bayesian update on the belief. The next action is then planned from the new belief.

When using an LLM to solve the POMDPs described above, we evaluate its ability to (1) serve as the planner, mapping belief to action, and (2) update its belief based on the history. For both assembly and troubleshooting, the task is described in the prompt. The reward structure and goal is mentioned as well. The action format is given. The history of actions and observations is also given. At each time step, the LLM needs to consider the history in the prompt to infer the current belief and plan an action that is likely to achieve the final goal. We measure the final reward obtained after termination using the reward models described.

### 3.2 LLMs for assembly and troubleshooting

In this section we describe how we use LLMs as decision-making agents that iteratively interact with the environment to solve object assembly and troubleshooting (described as POMDPs in the previous section) under uncertainty. The simulation is set up to assume that the LLM gives its planned instructions to another agent that is capable of executing the instruction (e.g., a person that the LLM agent is assisting or a robot with the requisite manipulation skills) and reports back with the observation. The LLM agent uses the observation to update its understanding of the problem and plan accordingly. A planning loop is set up where, at each time step until termination, the LLM is called with a prompt containing the following information:

#### 3.2.1 Task description

Defines the task and the role of the LLM as an interactive assistive agent. It instructs the LLM on the overall goal of the task, the reward module, and the information provided to the LLM to make a decision, such as the history, the object parts, and the functions of each part. It also specifies the output format.

#### 3.2.2 Parts

The list of parts in the object considered. The LLM is instructed to limit its answers to the parts present.

#### 3.2.3 Part functions

The functions of each part of the object and the function of the object as a whole.

#### 3.2.4 Status

The current status of the task (i.e., whether it is completed or not).

#### 3.2.5 History

The sequence of actions taken, and the corresponding observations received at each time step up until the current time stamp.

**Figure 1:**
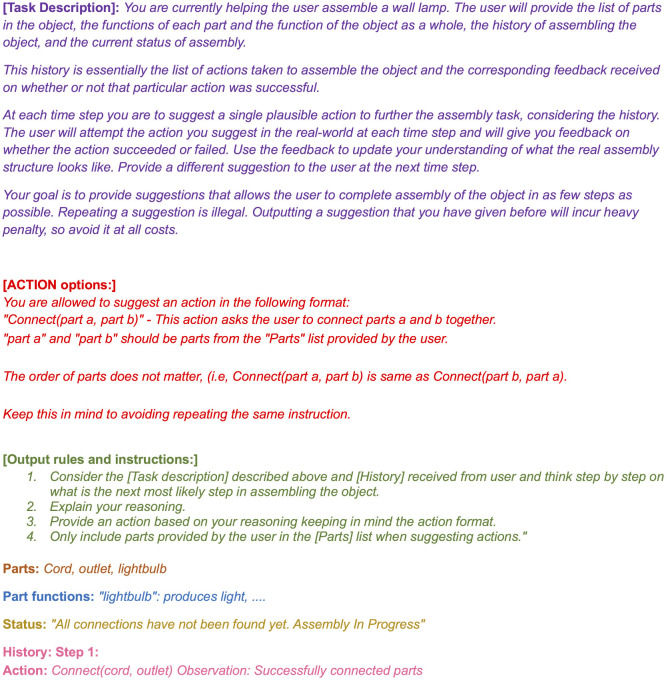
A sample prompt template for the LLM planner. The object parts and functions are for representational purposes and are different from the actual objects used.

Each call to the LLM has no memory of previous calls, so at each time step the history sequence is updated with the current action/observation pair, and the entire history is passed the next time the LLM is called. The planning loop is allowed to run for a maximum of n2 steps for assembly, and for a maximum of n2+n steps for troubleshooting for an object with *n* parts. These values correspond to the maximum number of pairwise connections for assembly and all possible error locations for troubleshooting. The planning template is shown in Figure 1.

### 3.3 LLMs integrated with a causal model

**Figure 2:**
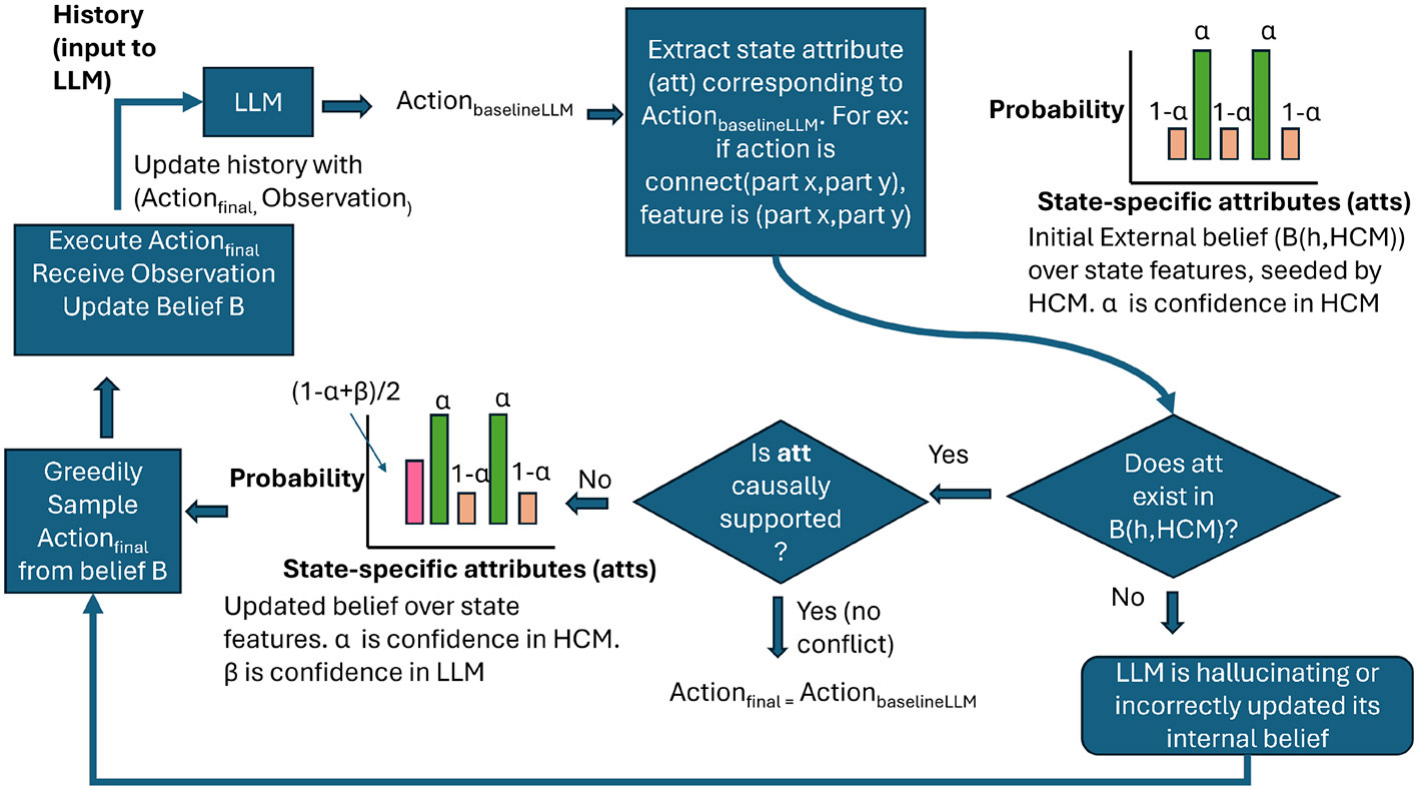
The overall methodology of supplementing LLM decision-making with human causal models for solving POMDPs.

We now describe our model for augmenting our baseline LLMs with a human causal mental model for improved decision making. We make the assumption that the human causal model of how an object functions serves as an approximate guideline for how someone would plan under uncertainty for downstream object-specific tasks such as assembly and troubleshooting. We propose to combine an LLM's decision together with a decision-making paradigm inspired by a human causal model to come to the final action planned at each time step. We hypothesize that supplementing our baseline LLM agents with a human causal model-guided framework will improve overall rewards for both tasks. The overall methodology is shown in Figure 2.

Our first step is to create a belief distribution over the task-specific system states that reflects the expert causal model. We follow a process similar to that described in (Basu et al. 2025) to achieve this. In the assembly task, the uncertainty arises from the fact that it is not known which parts should be connected to each other. The belief captures the probability at each time step of each possible pairwise part connection being present or not in the final assembly state. In the troubleshooting task, the uncertainty comes from not knowing which part or pairwise part connections are causing the observable malfunction. The belief is a probability distribution defined over all parts and part connections, capturing the probability that they could be the cause of the malfunction. The belief state of each POMDP is factored into separate distributions for state-specific features under an independence assumption. For assembly, the features include all pairwise part connections, while for troubleshooting, the features are all pairwise part connections and all object parts. That is,


$$\begin{array}{l} B\left(h\right)=\underset{att\in F}{\prod }P\left(att\in TS|h\right), \end{array}$$

where *B* is the belief, *att* represents state-specific features, *TS* is the true underlying state which is not fully observable, and *h* represents the history of actions and observations.

**Figure 3:**
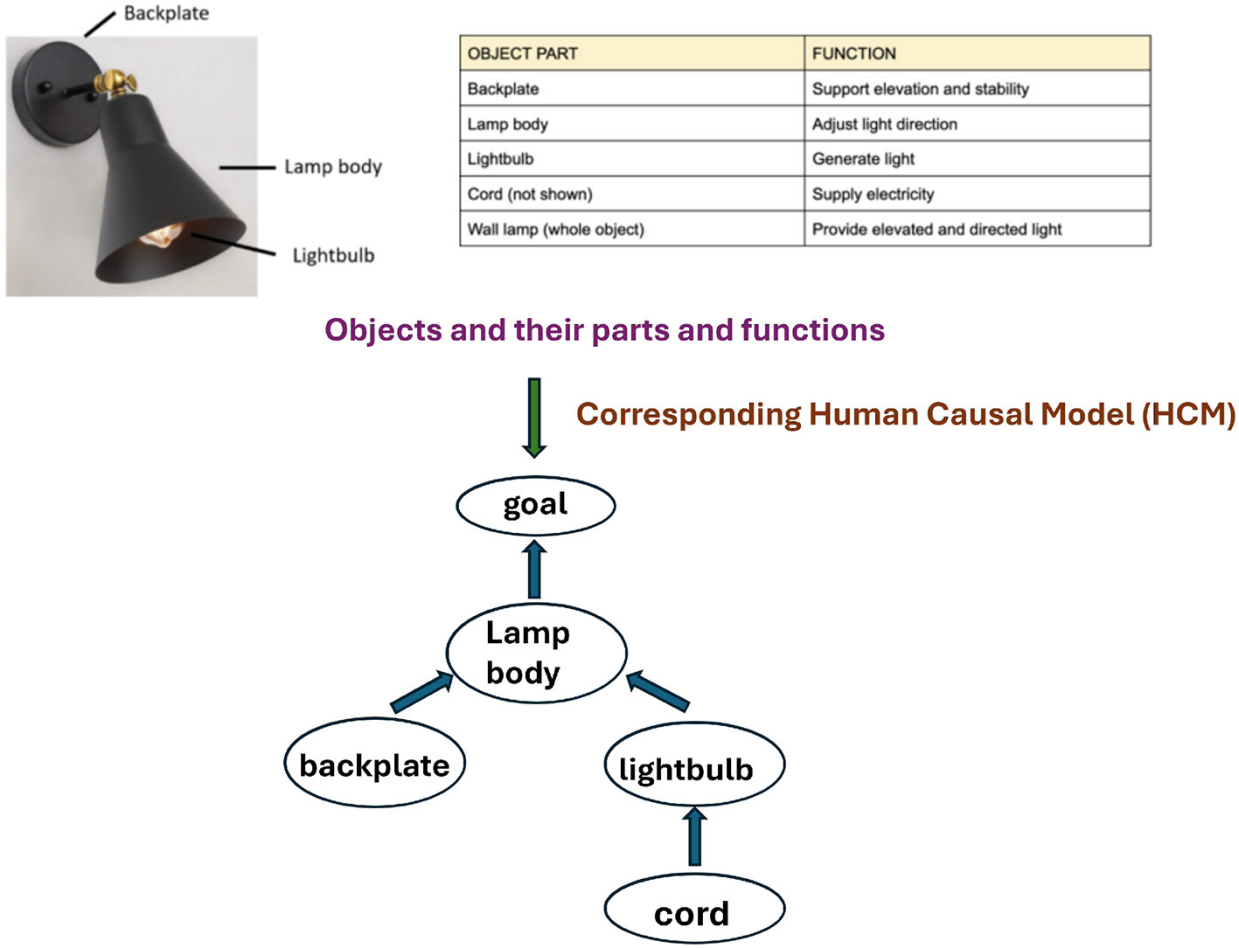
A sample object used in our study and the corresponding ground truth causal model for the object generated by our team.

The human causal model (HCM) is a directed graph where each node is an object part, and each edge denotes a causal link between the connected nodes. In our previous publications, we discuss in detail the elicitation of causal models from non-expert humans (Basu et al., 2025; Tatlidil et al., 2025). In particular, an object may elicit a range of causal models from different people depending on context and previous interaction with the object or some similar object. However, in this study, our causal models were determined directly by members of our research team, through expert knowledge found on the internet, and through mutual consensus. An example of a causal graph representing the human causal model of a wall lamp is shown in Figure 3.

The HCM is used to bias the belief distribution in a way that captures the human mental model such that,


$$\begin{array}{l} B\left(h,HCM\right)=\underset{att\in F}{\prod }P\left(att\in TS|h,HCM\right) \end{array}$$

Let *CA* represent the event that a state-specific attribute is also present in the HCM:


$$\begin{array}{l} \;P(att\in TS|h,HCM)=\\ P(att\in TS|h,CA)P(CA)+\\ P(att\in TS|h,CA^{c})P(CA^{c}), \end{array}$$

where *P*(*att*∈*TS*|*h, CA*) = α and *P*(*att*∈*TS*|*h, CA*^*c*^) = 1−α. Here α represents the probability that an attribute (part/connection) present in the expert causal model is present in the true assembly structure we are trying to discover or the contributing cause of the malfunction we are trying to remedy. It can be set by the user and implicitly captures our confidence in the human causal model.

The belief *B*(*h, HCM*) not only captures human preference over the state space, but also provides a direct one-to-one mapping between the belief and the action space due to the way the factoring was performed over the state space attributes. That is, in assembly, the belief over a pair of parts captures the probability that the pair is part of the assembly and also directly tells us that the corresponding action is to connect the two parts.

We assume that the LLM maintains its own belief distribution that we do not have access to, as well as its own internal policy mapping the history we input to an action. That is, *LLM*_*a*_ = *LLM*_π_(*B*_*llm*_(*h*)).

Once we obtain *LLM*_*a*_, we try to determine if the action aligns with the HCM-guided planning framework by first checking for conflicts. Conflicts arise in two cases:

#### 3.3.1 State space attribute corresponding to *LLM*_*a*_ is not found in belief *B*(*h, HCM*)

In this case, the LLM is suggesting actions that have already been tried before (and hence corresponding object parts have been filtered out in *B*(*h, HCM*)) or the LLM is hallucinating (e.g., suggesting an inspection of a part that does not exist in the object). In such cases, *LLM*_*a*_ is not a valid action, so we directly sample the attribute with the highest probability in *B*(*h, HCM*) and return the corresponding action as the final action: *Final*_*a*_ = *Greedy*_π_(*B*(*h, HCM*)).

#### 3.3.2 State space attribute corresponding to *LLM*_*a*_ is present in believe *B*(*h, HCM*) but not in the human causal model HCM

Let's call this instance *LLM*_*att*_. The action suggested by the LLM is a valid action; however, it is not one that is preferred by the expert. That is, *LLM*_*att*_ is not a feature present in the causal graph obtained from the expert. In such cases, we first update the probability of *LLM*_*att*_ in *B*(*h, HCM*) to reflect the LLM's preferences. Initially,


$$\begin{array}{l} B\left(LLM_{att}\right)=P\left(LLM_{att}\in TS|h,{CA}^{c}\right)\\ \;=1-\alpha \end{array}$$

Let us assume the confidence in the LLM's answer is β. The probability of the attribute is updated to have a value that is the average of the human's and the LLM's confidence. That is, *B*(*LLM*_*att*_) = (1−α+β)/2. Now we sample the attribute with the highest probability in the updated belief and return the corresponding action as the final action. Mathematically, we express this as *Final*_*a*_ = *Greedy*_π_(*B*(*h, HCM, LLM*)).

The value of β can be directly obtained from the log probabilities of tokens in the LLM's answer or set to a predefined value by the user. If no conflict arose, then the LLM suggested an action that aligned with the human causal model. In such cases : *Final*_*a*_ = *LLM*_*a*_.

The value of *Final*_*a*_ is the action that is executed in the environment, and together with the observation received, it is appended to the history *h* to plan the next step. The belief maintained (*B*) is also filtered after the new action and observation are received, before the next step is planned.

## 4 Experimental results

### 4.1 Experimental setup

This study builds upon our previous studies published in (Basu et al. 2025), and uses the same task formulation. The data used to run the experiments—including the object specifications, ground truth assembly, and troubleshooting conditions of these objects, and the human causal models—is the same data used in (Basu et al. 2025) and a subset of the data published in our prior study (Tatlidil et al., 2025). We provide the data used in the Supplemental Material for better understanding. The experimental setup was done in the same way as described in (Basu et al. 2025), but we give a short overview here for completeness.

The ground truth assembly plans were chosen through careful discussion and study of assembly manuals available online. The planner is tasked with discovering the ground truth assembly plan in as few steps as possible. For troubleshooting experiments, we chose one observable malfunction that the planner had to troubleshoot—for example, for the wall lamp, it was “No light being produced”, for the bicycle, it was “Back wheel not spinning”. Troubleshooting manuals were consulted to come up with all potential causes of the chosen observed malfunction, which served as the ground truth for troubleshooting experiments. The planner is tasked with discovering the true cause of the malfunction in as few steps as possible. For each object, we run the troubleshooting planner against each ground truth error location and report the average reward, because the performance of the planner should reflect whether or not it could effectively troubleshoot a problem, no matter what the source was.

We use the same data and setup to obtain a fair comparison to the methodology of (Basu et al. 2025) that embeds crowd-sourced and expert models into the belief state of the assembly and troubleshooting POMDPs prior to solving them with an online planning algorithm, POMCP (Silver and Veness, 2010). The rewards reported in (Basu et al. 2025) for solving the POMDPs without any prior and with expert human causal models as priors serve as the two baselines to which we compare our LLM-based planners. We evaluated assembly and troubleshooting for seven different objects of varying complexity: *wall lamp, desk lamp, flashlight, kerosene lamp, bicycle, sink*, and *toilet*.

### 4.2 Model selection and hyperparameter settings

We use the prompting template introduced in Section 3.2 to test the abilities of different LLM models to solve our assembly and troubleshooting POMDPs. We use LLM models GPT-4o (OpenAI, 2024a), o3-mini (OpenAI, 2025b) and o4-mini (OpenAI, 2025a). GPT-4o is a flexible, flagship model from OpenAI that does not explicitly use reasoning tokens but is a multi-purpose GPT model with a context window of 128,000. The o3-mini and o4-mini models have been trained using reinforcement learning to perform chain-of-thought reasoning and explicitly use reasoning tokens. They have a larger context window of 200,000 and more recent knowledge cutoff dates than GPT-4o. For GPT-4o, we set the temperature parameter to 0.7 in API calls. For o3-mini and 04-mini, the reasoning effort was set to “high” for all experiments.

The value of α, which captures confidence in the human causal model, is set to 0.9 for all experiments. The value of β, which captures confidence in the LLM's answers, is set to 0.7 for o3-mini and 04-mini. This value can be set by the user. We assumed that we should assign a confidence to the LLM that is less than what we assigned to the human causal model; hence, we settled on 0.7. Experimenting with how different confidences in the human and LLM answers affect planning performance is left to future study. For GPT-4o, we could access the log probabilities of the tokens — hence the probability in the LLM's answer was directly derived by taking an average across all token probabilities in its answer.

### 4.3 Results for assembly task

**Figure 4:**
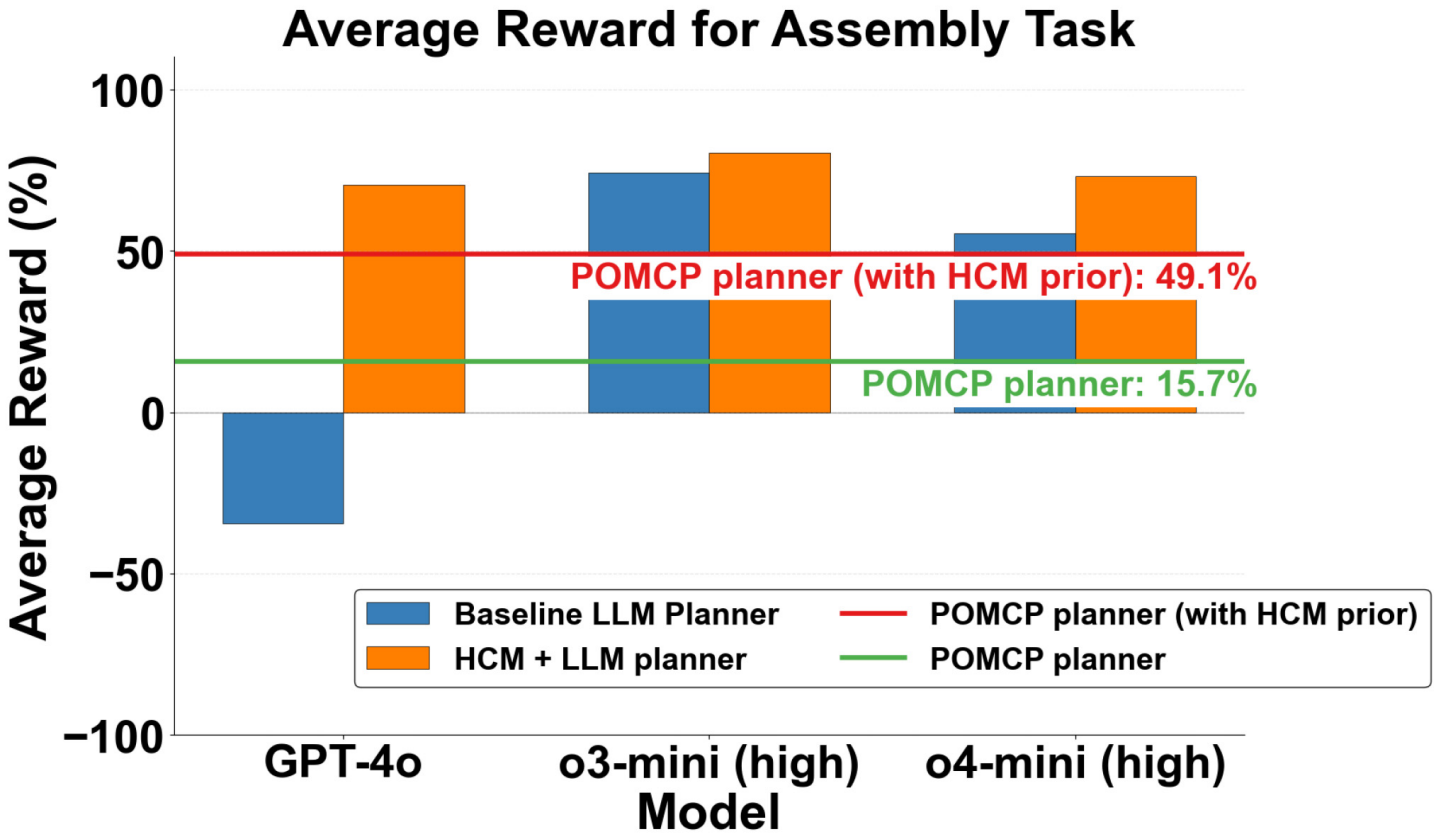
Average reward (as a % of maximum achievable reward) across all objects. Higher is better.

**Figure 5:**
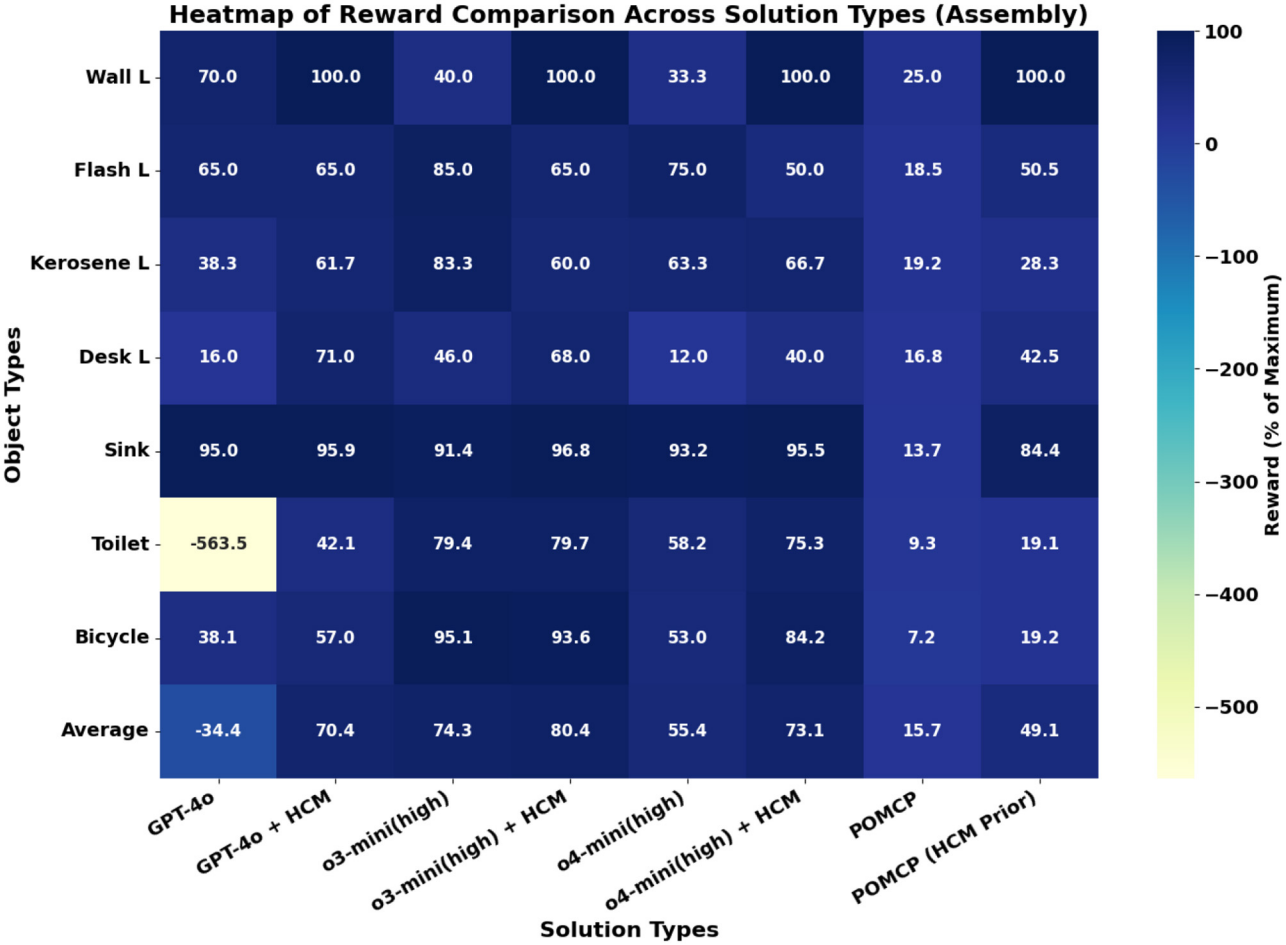
Heatmap for assembly rewards for objects as % of maximum achievable reward for Baseline LLM and non-LLM planners and their human causal model (HCM) augmented counterparts. For each LLM model tested, we report the reward obtained by using them directly as planners (model name on x-axis) and the reward obtained when augmenting them with causal models (“model + HCM” on the x-axis). For non-LLM planners, we report results from study (Basu et al., 2025), where POMCP was used to solve the POMDPs without any prior (“POMCP”) and then with a human causal model as prior (“POMCP (HCM prior)”).

The average reward values across all objects obtained from different planners for assembly are shown in Figure 4. A breakdown of object-wise rewards across different planners is shown in Figure 5. For each object, the reward for LLM-based planners is the average reward obtained across 10 runs of the corresponding planner. It can be seen in Figure 4 that the baseline o3-mini and o4-mini planners outperform both non-LLM planners. The GPT-4o baseline planner suffered from inaccurate belief updates, especially for complex objects like a toilet (see Figure 5), which resulted in many repeated instructions that incurred penalties. This caused it to underperform even the POMCP planner, which had no informative priors and hence is expected to perform the worst. Our causally augmented LLM planners always outperform their baseline LLM planner counterparts, with an average improvement in rewards over the baseline of 305%, 8.2%, and 32% for GPT-4o, o3-mini, and o4-mini variants, respectively.

### 4.4 Results for troubleshooting task

**Figure 6:**
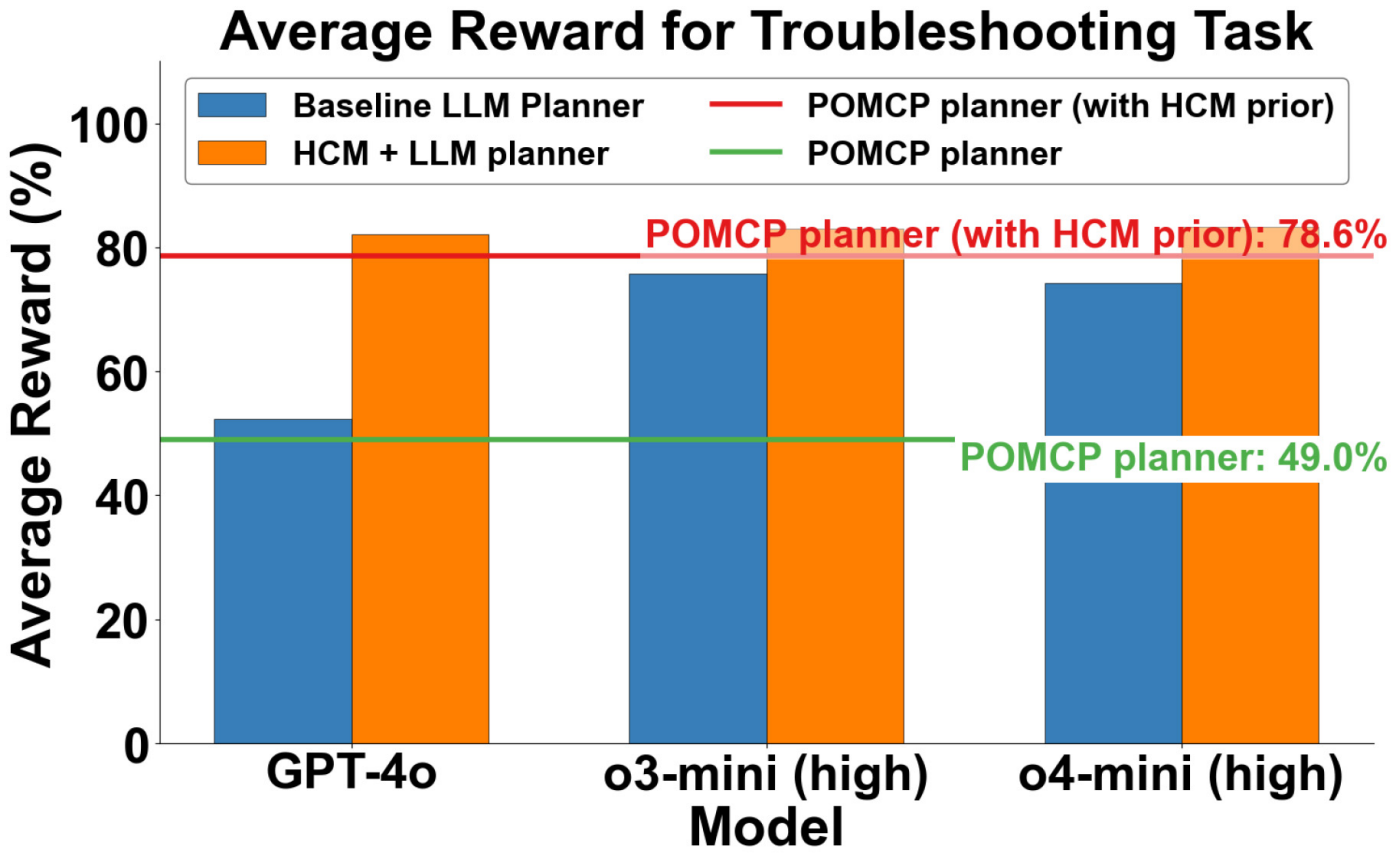
Average reward (as a % of maximum achievable reward) across all objects. Higher is better.

**Figure 7:**
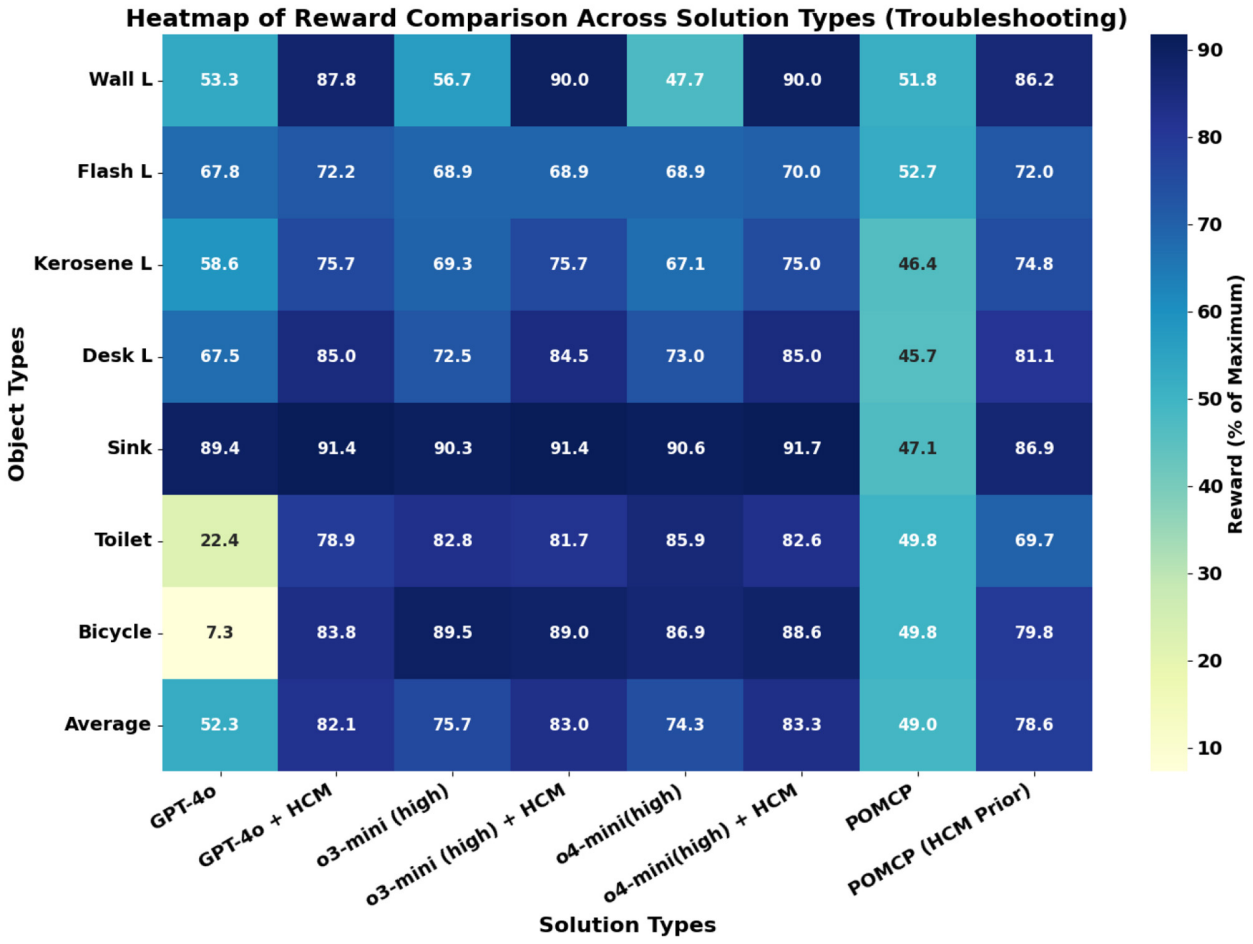
Heatmap for Troubleshooting rewards for objects as % of maximum achievable reward for Baseline LLM and non-LLM planners and their human causal model (HCM) augmented counterparts. For each LLM model tested we report the reward obtained by using them directly as planners (model name on x-axis) and the reward obtained when augmenting them with causal models (“model + HCM” on the x-axis). For non-LLM planners, we report results from study (Basu et al. 2025), where POMCP was used to solve the POMDPs without any prior (“POMCP”) and then with a human causal model as prior (“POMCP (HCM prior)”).

The average reward values across all objects obtained from different planners for troubleshooting are shown in Figure 6. An object-wise breakdown is shown in the heatmap in Figure 7. The reward for each object shown in Figure 7 is the average of the reward obtained from solving for each potential error location of a particular malfunction. The reward for each error location is reported as the average reward obtained across 10 runs of the LLM planners. On average, the baseline LLM planners outperform the POMCP planner rewards but underperform the POMCP with a human causal prior. The causal model augmented LLM (LLM + HCM) planners always outperform their baseline LLM planner counterparts, with an average improvement in rewards over the baseline of 57%, 9.6%, and 12% for GPT-4o, o3-mini, and o4-mini variants, respectively. The LLM + HCM planner also outperforms POMCP with a causal prior.

### 4.5 Failure modes in LLMs and how they were improved with human causal models

**Figure 8:**
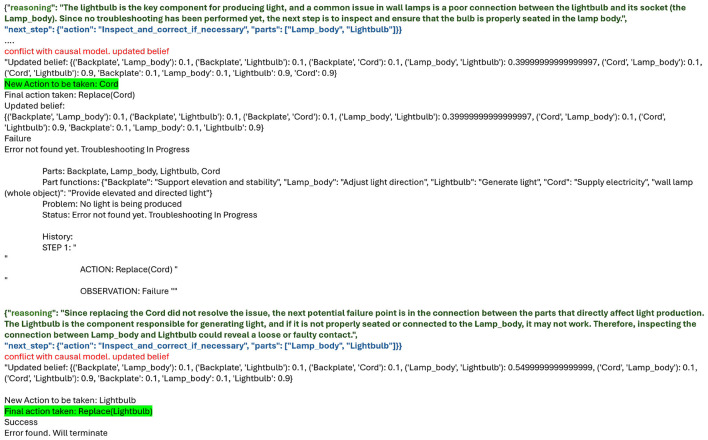
Failure mode example: o3-mini showing inability to abstract. Assumes the presence of a socket (see “reasoning” provided by the LLM on top in green) without considering the actual part functions provided.

**Figure 9:**
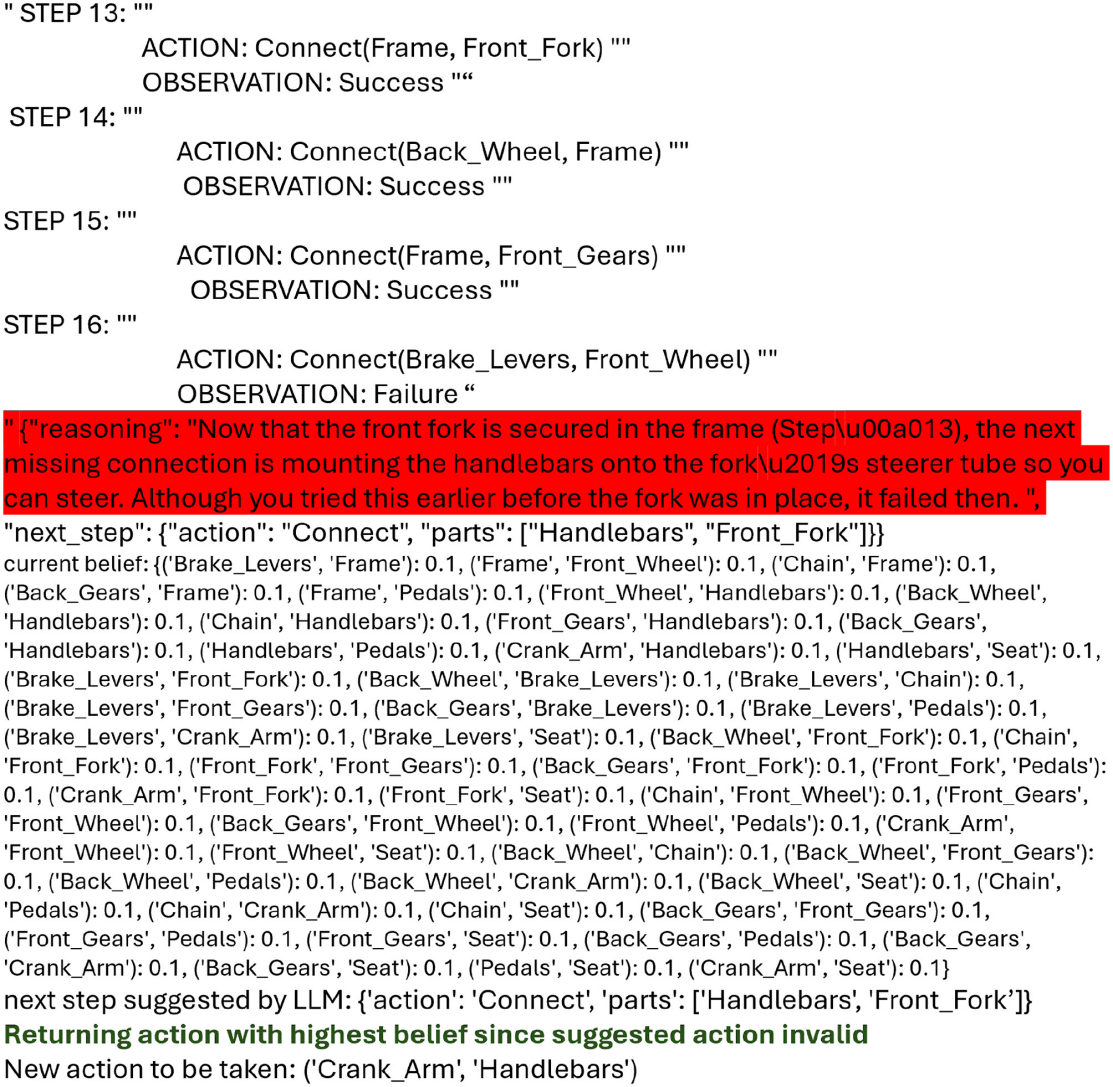
o4-mini exhibiting overthinking to bypass prompt instructions, highlighted in red.

While we observe a general trend showing improvement on adding causal models, we qualitatively examine the explanation generated by LLMs to gain insights into their failure modes. In our prompting template, we had asked the LLM to give a “reasoning” for its decision. It should be clarified that this rationale that it presents is part of the required output and not the same as the “reasoning traces” or intermediate series of tokens it generated prior to giving us the output. The intermediate “reasoning tokens” for o3-mini and o4-mini are hidden by OpenAI:

1. **Inability to abstract to a particular instance of the object based on the information provided -** We look at the case of troubleshooting the wall lamp with o3-mini. In Figure 7, it can be seen that adding the HCM improved the rewards from 56.7% to 90%. In Figure 8, we examine the reasoning of the LLMs behind those decisions. The reasoning from the LLM (as shown in green in the logs in the “reasoning” section) is quite reasonable; however, it assumes the existence of a socket in the lamp body and hence considers inspecting the connection between the lightbulb and the lamp body as a plausible step in troubleshooting. While in general it is extremely common to have a socket to which we fix our lightbulb, the wall lamp design we are working with assumes a simplified model where there is no socket and the lightbulb is expected to be directly connected to the cord. The function of the “lamp body” is mechanical—it is to adjust the direction of light. While we mention the parts and part functions in the prompt, the LLM ignores these definitions and falls back on its default understanding of a wall lamp to troubleshoot it.
2. **Inability to correctly perform belief updates—**Correctly updating the belief of the world is crucial to efficient planning in POMDPs and an important step in classical POMDP planners. The LLM planner, powered by older models such as GPT-4o, often exhibited behavior that indicated it failed to correctly update the belief based on the history of actions and observations taken. This inability often manifests as repeated instructions despite clear instructions not to repeat anything that the user has tried before. While this behavior seemed to have been corrected in o3-mini, we observed it again in o4-mini. Supplementing with the HCM was helpful in such situations, as it prevented providing inaccurate instructions, which incurred penalties and reduced cumulative rewards.
3. **Overthinking - inability to follow instructions—**We noticed certain instances where the LLM model “overthinks”, and essentially comes up with a rationale to bypass user-instructions given in the prompt. We noticed several instances where o4-mini explains why it repeats instructions that were strictly forbidden in the prompt (see Figure 9 where the reasoning generated by the LLM is highlighted in red). While this can be useful in certain cases, for the tasks we tested, it incurred penalties and had the same effect as weaker models that failed to follow instructions.

## 5 Discussion and conclusion

In this article, we investigate whether LLMs can serve as effective interactive planners in object assembly and troubleshooting under uncertainty, and if augmenting LLMs with human causal models can improve cumulative rewards obtained. LLMs are currently divided into two types: non-reasoning models, which are general-purpose models without explicit reasoning capabilities, and reasoning LLM models, which have been enabled with reasoning abilities either at training or at inference time. With the advent of reasoning LLMs, there is a growing body of research on improving the general reasoning capabilities of LLMs, which in turn improves their overall performance across different tasks. These techniques, however, do not focus on teaching LLMs to explicitly reason causally. Rather, they depend on teaching them to identify correct chains of tokens out of the many possible chains they can generate, through explicit training or external verifiers. Inspired by human cognition that is characterized by causal model building to aid planning under uncertainty, we hypothesize that if human causal models can be used to weigh an LLM's decisions, it would allow us to leverage the LLM's world knowledge in a causally meaningful manner for both reasoning and non-reasoning LLMs.

We demonstrated that by maintaining an external probability distribution over system states, we can improve upon just using LLMs as policy. In our case, the probability distribution is initially biased using a causal model and updated at each time step to reflect the joint preferences of the LLM and human. In case of conflicts, our hybrid reasoning framework always cedes to human causal preferences. The way the belief update works, an LLM-suggested action that is not supported by the causal model will only be executed if all causally supported actions are exhausted or if the LLM suggests the same action enough times that the probability surpasses the confidence given to causal actions. In future studies, we hope to explore how rewards would be affected if we gave varying confidence to LLMs and human causal models depending on the particular task or environmental conditions. We also hope to explore more nuanced prompting techniques to improve our baseline LLM results. Moreover, we hope to explore how human causal models can be used to guide inference time scaling to improve rewards without explicitly having to maintain a belief distribution.

Mental causal models share with Causal Bayes Nets (Pearl, 2009) that they represent the causal structure that relates causes to effects, but they are incomplete and tend to be qualitative (Sloman, 2022). We elicited such qualitative causal models using the only method that has shown success in empirical evaluation (Tatlidil et al., 2025). Although we used expert knowledge to build the causal models in our current study, expertise is not necessary for this approach to succeed. (Tatlidil et al. 2025) shows that causal models of objects can be obtained from laypeople using multiple methods, such as by asking counterfactual questions or by simply asking people to draw causal graphs with minimal instruction. (Basu et al. 2025) shows that even imperfect causal models obtained from laypeople can be used to improve algorithm performance on assembly and troubleshooting tasks. Furthermore, (Liefgreen and Lagnado 2023) demonstrates that laypeople can generate causal models of legal arguments, suggesting that eliciting causal models is feasible across different domains. To summarize, prior study suggests that non-experts can generate causal models across different domains, and their models can be used to improve robot planning algorithms.

Given the rapid advancement of LLMs, one may wonder if keeping humans in the loop will continue to be necessary for very long. We argue that human involvement will continue to be essential for tasks that rely on causal knowledge. LLMs are not able to represent and deliberate on symbolic material, which is vital for causal learning and reasoning. Indeed, recent literature suggests that state-of-the-art LLMs continue to struggle with causal inference and reasoning tasks. (Miliani et al. 2025) showed that LLMs struggle even with a very simple causal reasoning task that involved determining the cause-and-effect relationship between two events; they tend to confuse temporal knowledge with causal knowledge and heavily rely on the linguistic order of events. (Jin et al. 2024) evaluated LLMs' ability to infer causality from correlational relationships and found that simply paraphrasing event descriptions with semantically equivalent descriptions can reduce the F1 score on this task as much as 0.39. Finally, (Saklad et al. 2025) demonstrated that when LLMs are used to infer causality from more realistic text involving more than just two variables, even the best performing model only reaches an F1 score of 0.48. Moreover, in our current study, we focused on relatively simple everyday objects that most people have some understanding of how they work. Combined with the previous study demonstrating that HCMs for such objects can be elicited using methods that are easy to implement, we believe our hybrid-reasoning framework will continue to be more favorable over using LLMs alone until there is substantial improvement in the ability of LLMs to learn and reason with causal knowledge.

## 6 Future work

This study opens up a range of directions for future study. First, future studies should address several key limitations of this work. We did not test the framework with different confidence values in the LLM's answers. In certain cases, such as in assembly, it is likely that the causal model is not as helpful because it represents functional relationships, not structural ones. In such cases, it probably makes sense to trust the LLM's answers as much as the causal model. Moreover, we do not test different ways of assigning confidence to the LLM's answers and the human, but make the assumption that an expert causal model is always to be trusted more than any LLM. In the future, we want to explore the effect of different confidence values in the LLM and the human on the overall reward.

We also do not test the ability of the LLM to generate causal models. Instead, we try to use the LLM as policy directly. However, the question that remains unanswered is whether using the LLM as policy is better than using the LLM to extract causal models and then using those as priors in POMDPs. Considering the recent advances in reasoning LLMs, it would make for an interesting future article to evaluate how accurate LLM-generated causal models are compared to human-generated ones using the same extraction technique. We also do not consider evolving or dynamic causal models where the human-generated causal model is allowed to evolve with observations from the environment or information from the LLM. In the future, we hope to explore the use of evolving causal models for more flexibility in decision-making than is afforded through the static causal models that we have explored in this study. We also hope to explore different prompt-engineering techniques to refine our prompts to elicit better answers. Moreover, though, our study shows that LLMs, while powerful, remain fundamentally limited and need to be buttressed with causal knowledge originating from human experts.

## Data Availability

The original contributions presented in the study are included in the article/Supplementary material, further inquiries can be directed to the corresponding author.
