# Supplementary material for: Augmenting large language models with psychologically grounded models of causal reasoning for planning under uncertainty
Source: Front Artif Intell. 2026 Jan 30;8:1730614. doi: 10.3389/frai.2025.1730614 (PMC12901353; doi:10.3389/frai.2025.1730614)
Supplement: Supplementary file 1 [file Data_Sheet_1.docx]

**Supplementary materials for “Augmenting Large Language Models with**

**Psychologically Grounded Models of Causal**

**Reasoning for Planning Under Uncertainty”**

**For all 7 objects used, we provide the object diagrams, parts and functions, the ground truth causal model we developed, the ground truth plan of assembly, and the troubleshooting conditions.**

**The diagrams, part/function tables, the ground truth causal models and the troubleshooting conditions for all objects used in the experiments.**

**Figure 1**


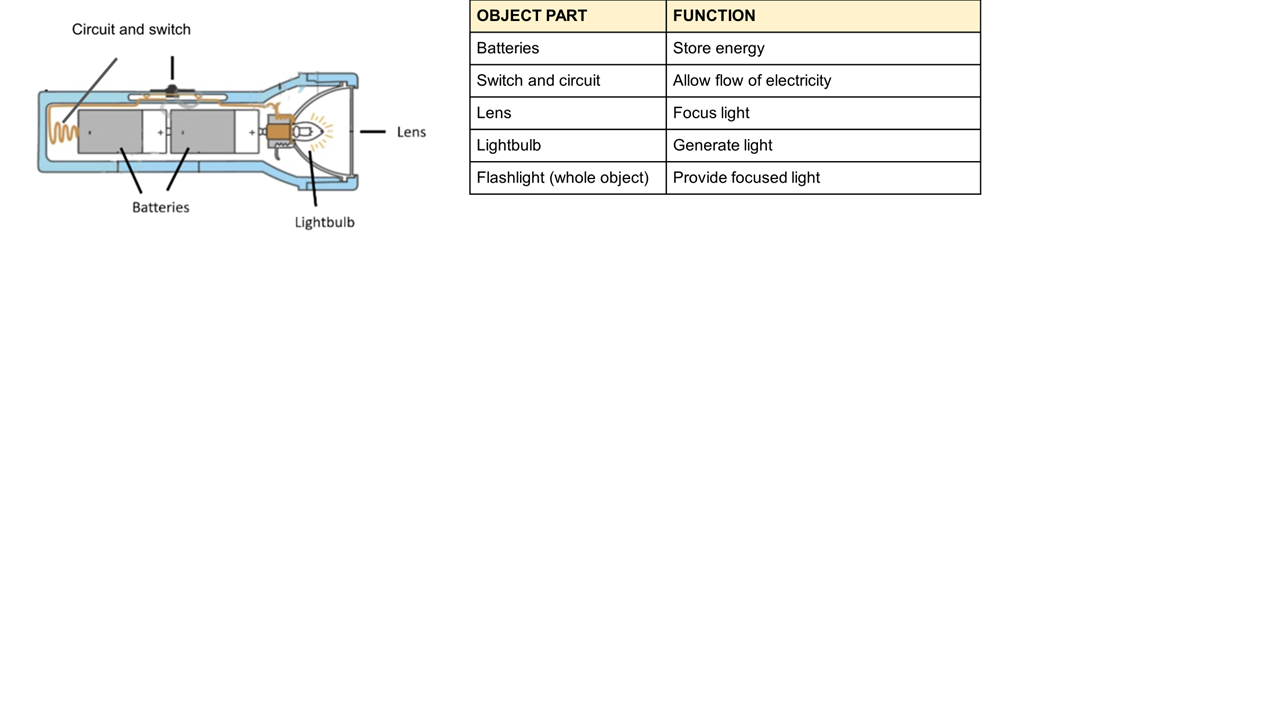
The diagram, part/function table, and the ground data for the flashlight.

**Ground truth causal model:**

**
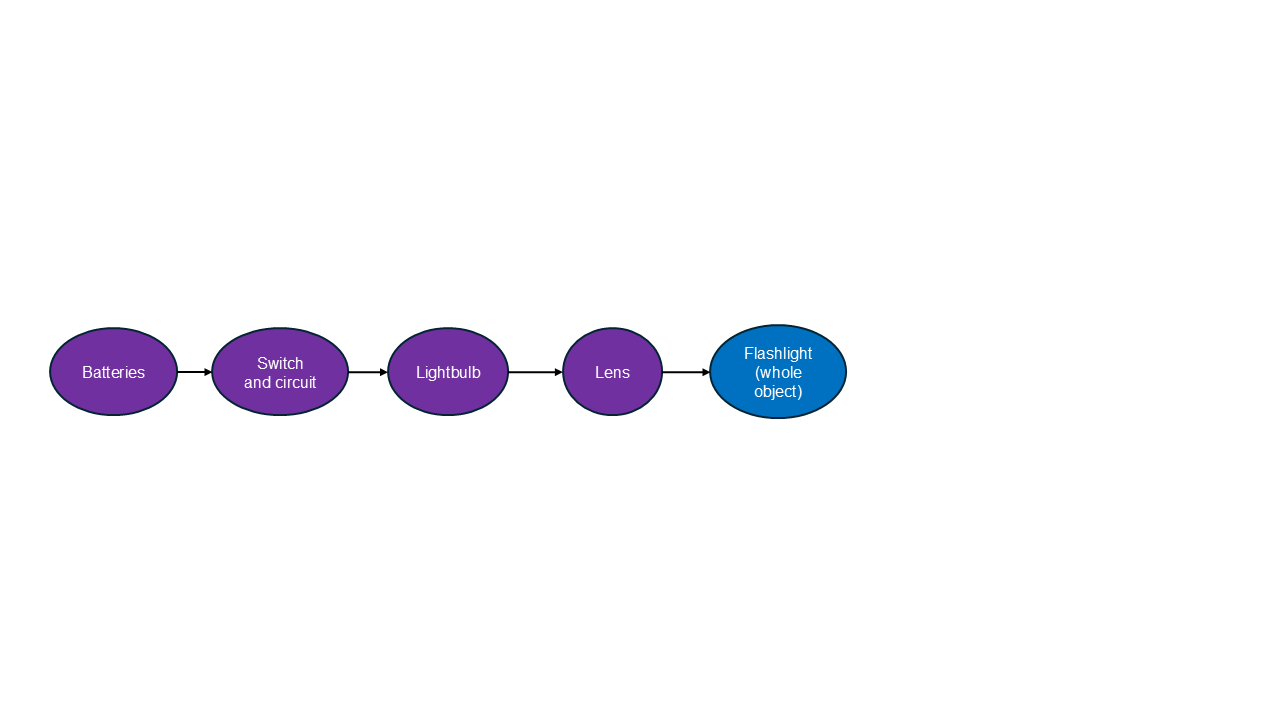
**

**Ground truth assembly plan as part connections:**

("Batteries", "Switch_and_Circuit"), ("Batteries", "Lightbulb"), ("Switch_and_Circuit", "Lightbulb"), ("Lens", "Lightbulb")

**The problem selected for troubleshooting:** "No light is being produced.”

**Potential ground truth error locations that could have caused this problem:**

Malfunctioning connections between the following parts**:**

- - **("Batteries", "Switch_and_Circuit"),**
  - **("Batteries", "Lightbulb"),**
  - **("Switch_and_Circuit", "Lightbulb")**

Malfunctioning parts:

- - **"Switch_and_Circuit"**
  - **"Batteries"**
  - **"Lightbulb"**

**Figure 2**


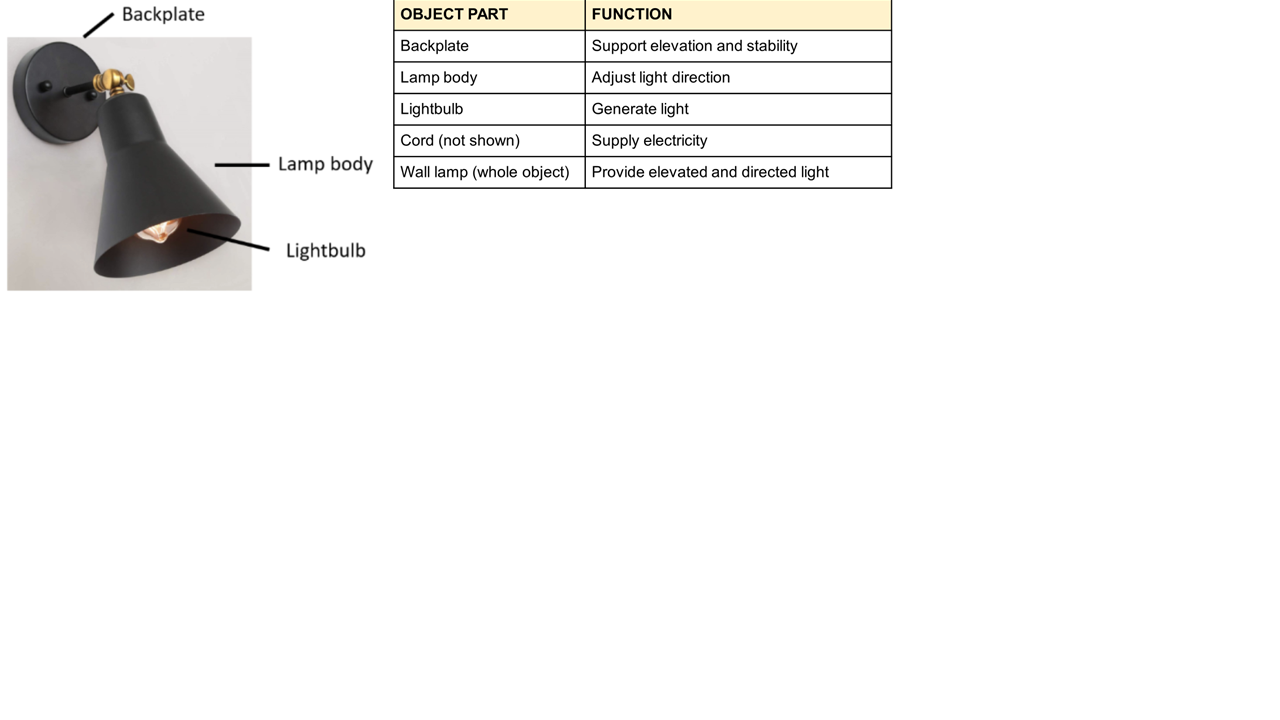
The diagram, part/function table, and the ground truth data for the wall lamp.


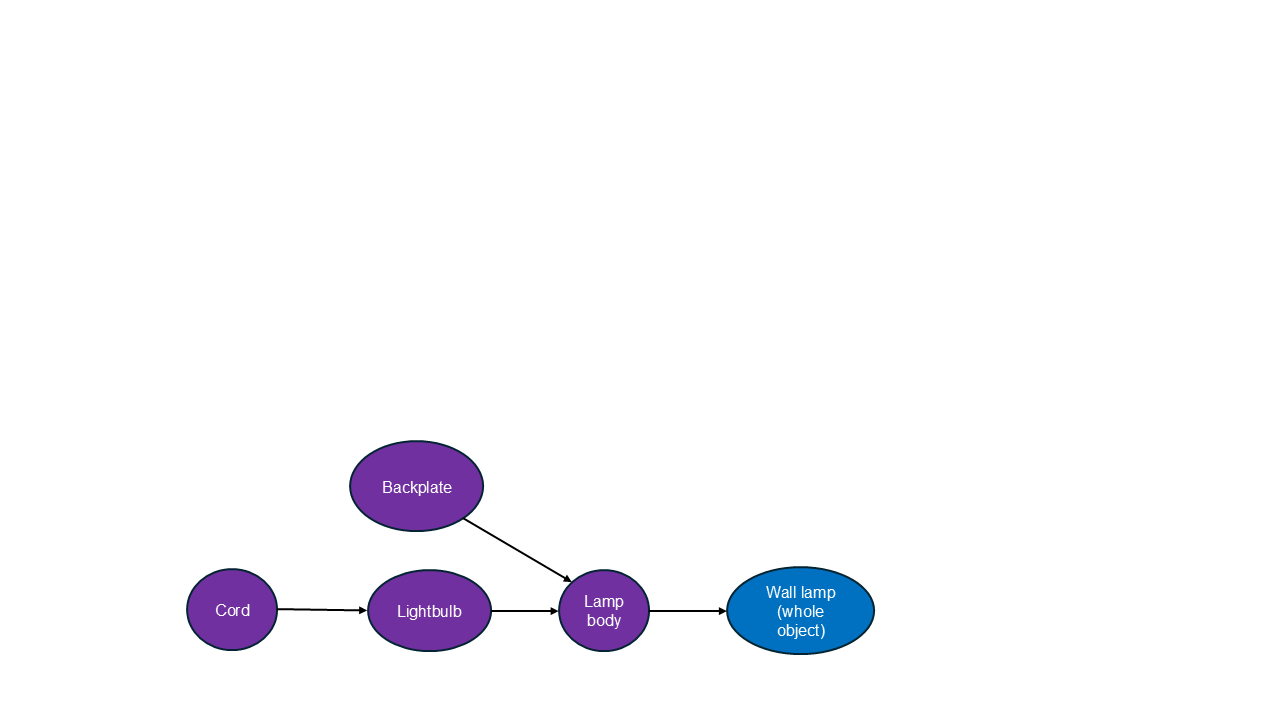
**Ground truth causal model:**

**Ground truth assembly plan as part connections:**

("Backplate", "Lamp_body"), ("Lamp_body", "Lightbulb"), ("Cord", "Lightbulb")

**The problem selected for troubleshooting:** "No light is being produced.”

**Potential ground truth error locations that could have caused this problem:**

Malfunctions connections between the following parts**:**

- **("Cord", " Lightbulb "),**

Malfunctioning parts:

- - **Lightbulb**
  - **Cord**

**Figure 3**


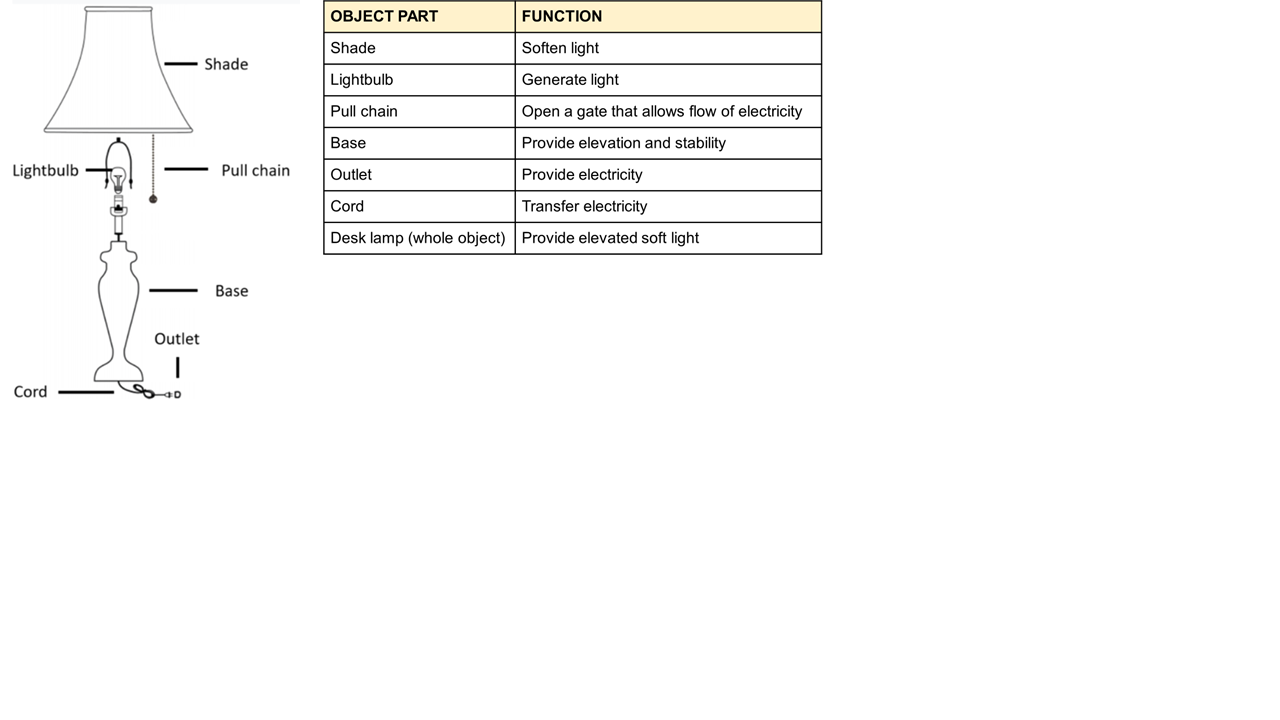
The diagram, part/function table, and the ground truth data for the desk lamp.

**Ground truth causal model:**
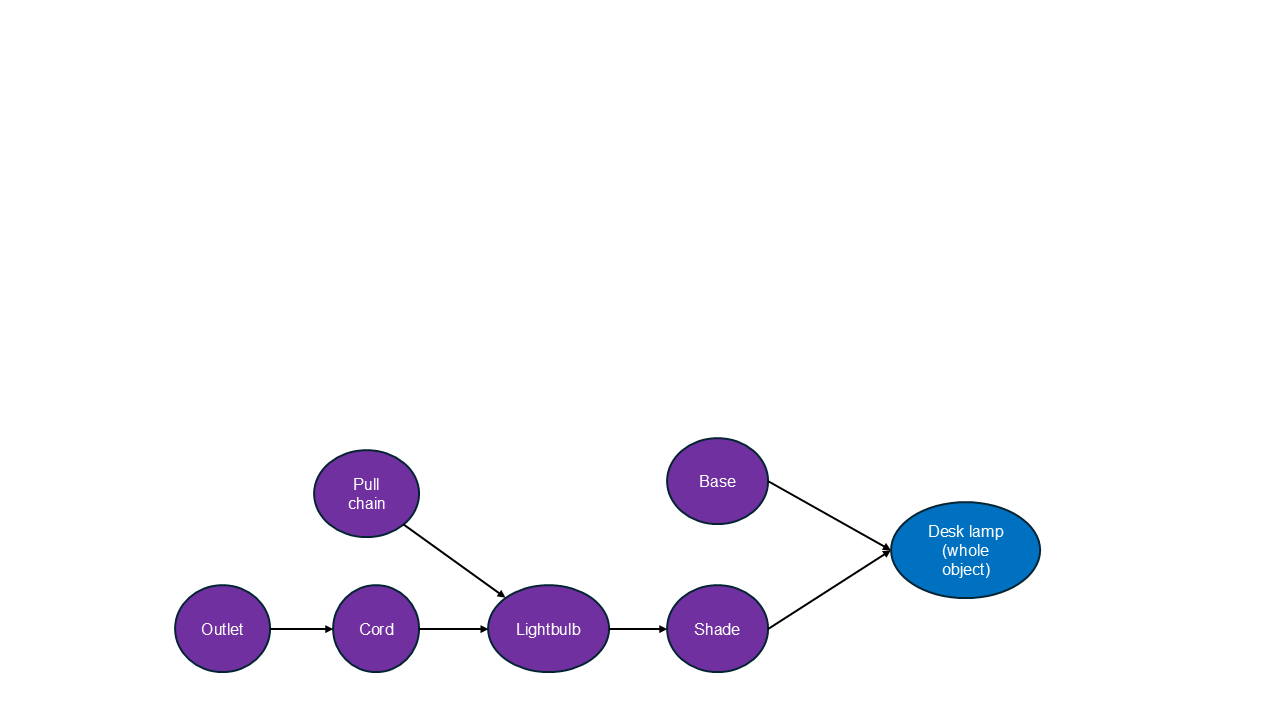


**Ground truth assembly plan as part connections:**

("Outlet", "Cord"), ("Cord", "Lightbulb"), ("Base","Lightbulb"), ("Pull_chain", "Lightbulb"), ("Shade", "Lightbulb")

**The problem selected for troubleshooting:** "No light is being produced.”

**Potential ground truth error locations that could have caused this problem:**

Malfunctioning connections between the following parts**:**

- **("Outlet", "Cord"),**
- **("Cord", "Lightbulb"),**
- **("Pull_chain", "Lightbulb"),**

Malfunctioning parts:

- **Lightbulb**
- **Pull_chain**
- **Outlet**
- **Cord**

**Figure 4**


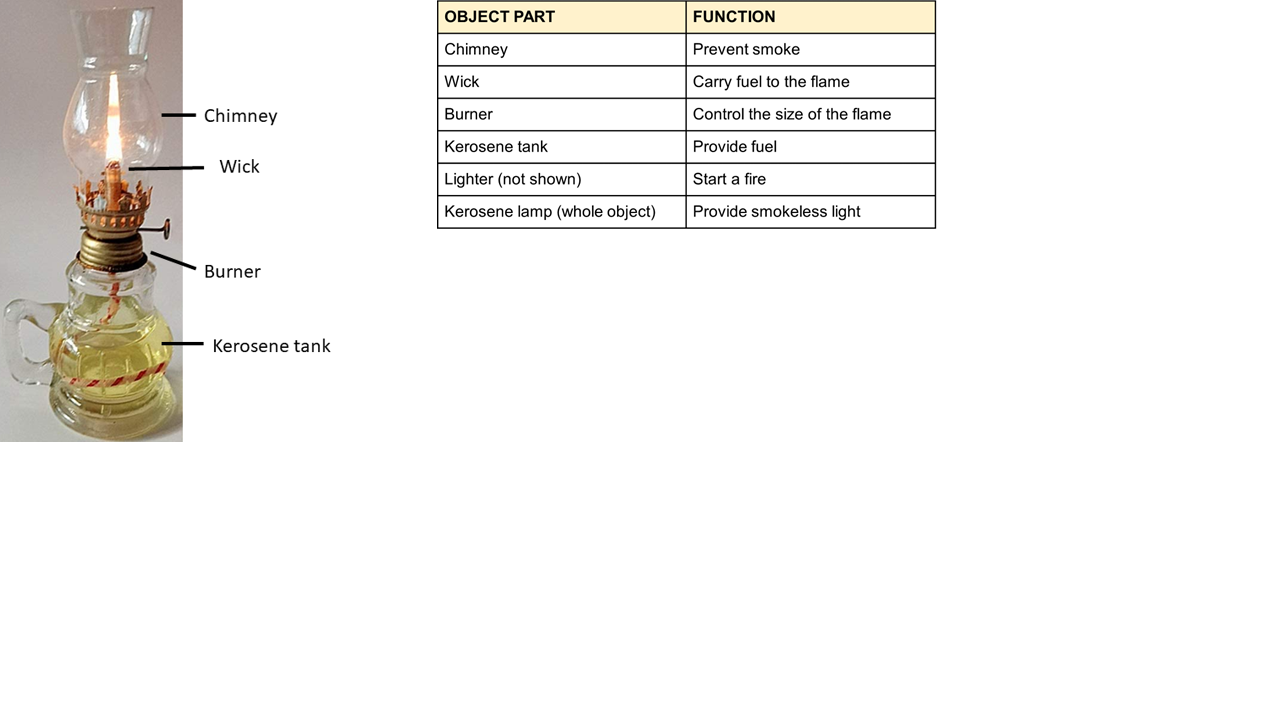
The diagram, part/function table, and the ground data for the kerosene lamp.


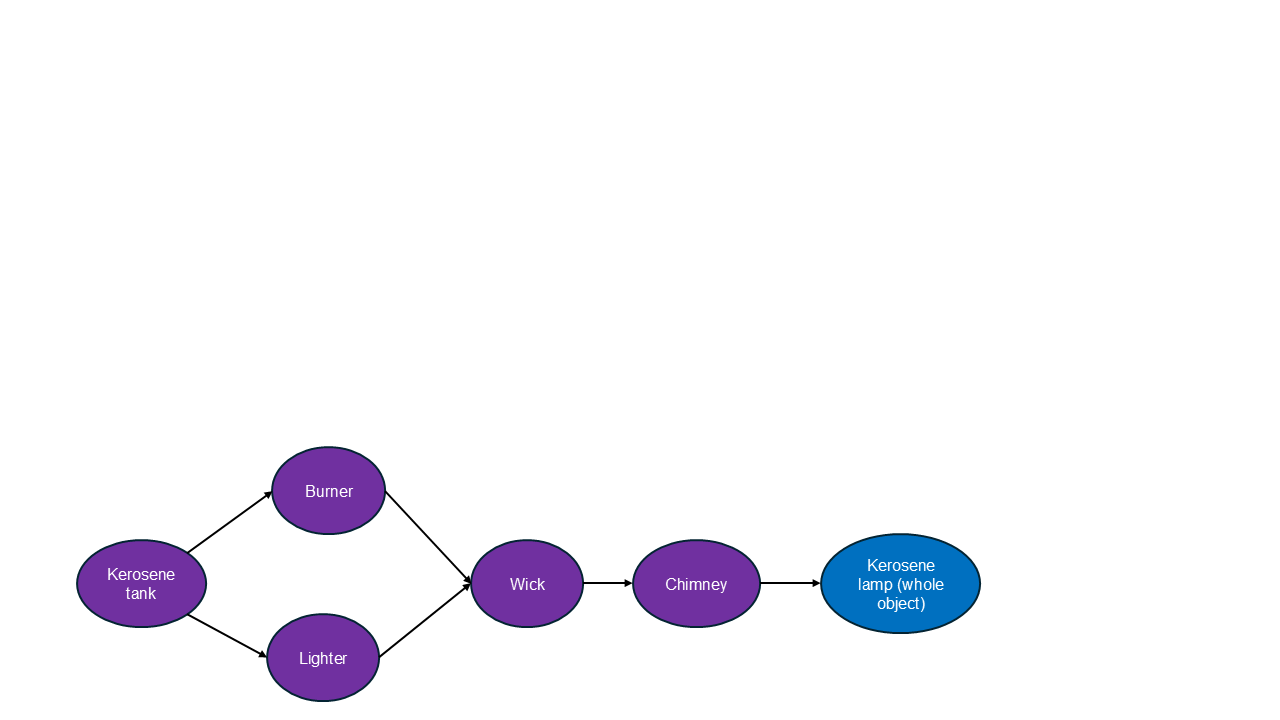
**Ground truth causal model** :

**Ground truth assembly plan as part connections:**

("Kerosene_Tank", "Burner"), ("Burner", "Wick"), ("Lighter", "Wick"), ("Chimney", "Burner")

**The problem selected for troubleshooting:** "No light is being produced.”

**Potential ground truth error locations that could have caused this problem:**

Malfunctioning connections between the following parts**:**

- - **("Kerosene_Tank", "Burner"),**
  - **("Burner", "Wick"),**
  - **("Lighter", "Wick"),**

Malfunctioning parts:

- **Kerosene_Tank,**
- **Burner**
- **Lighter**
- **Wick**

**Figure 5**


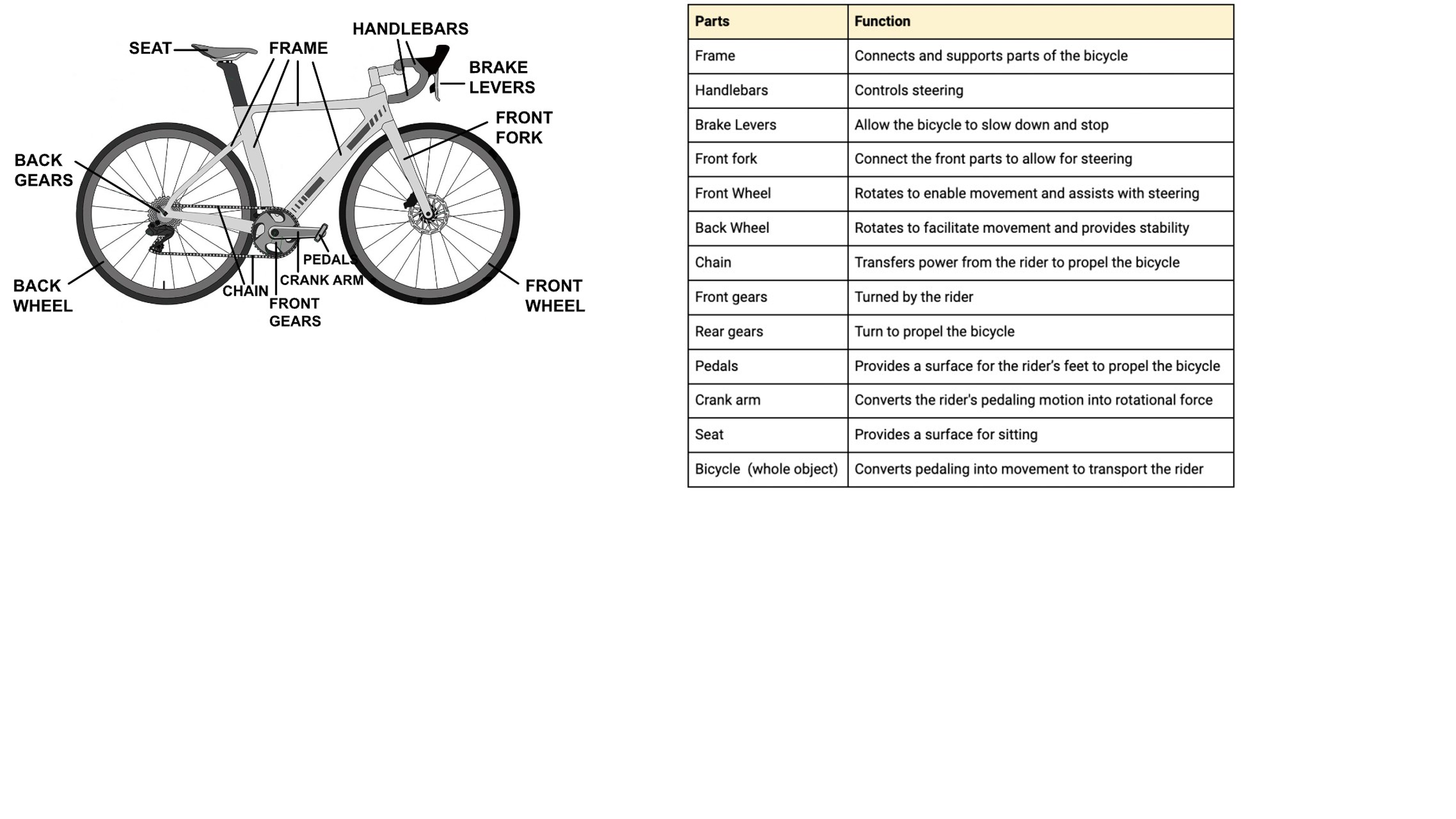
The diagram, part/function table, and the ground truth data for the bicycle.

**Ground truth causal model:**


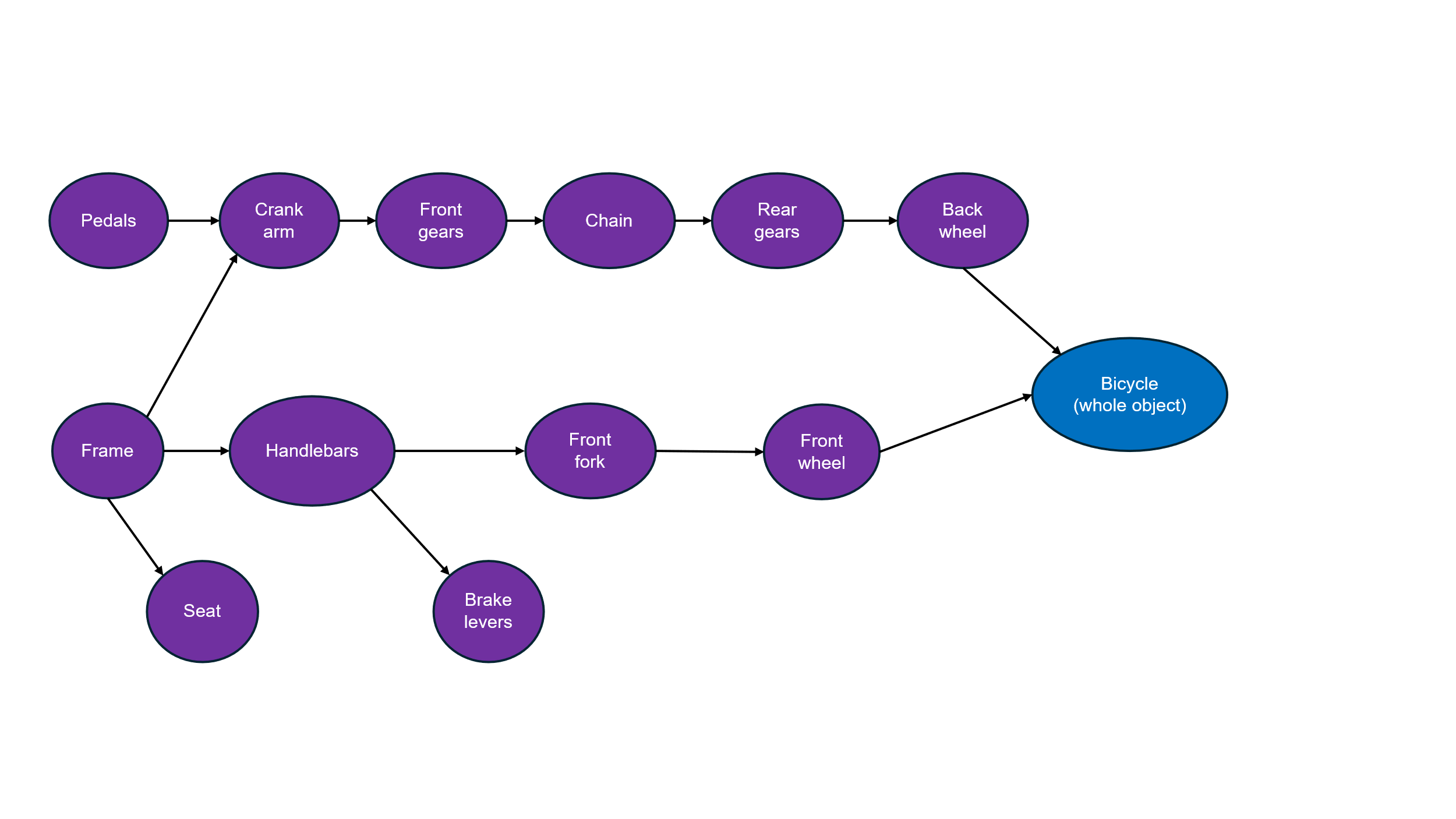


**Ground truth assembly plan as part connections:**

("Frame", "Handlebars"),

("Handlebars", "Brake_Levers"),

("Frame", "Front_Fork"),

("Front_Fork", "Front_Wheel"),

("Frame", "Seat"),

("Frame", "Back_Gears"),

("Front_Gears", "Frame"),

("Pedals", "Crank_Arm"),

("Crank_Arm", "Front_Gears"),

("Front_Gears", "Chain"),

("Back_Gears", "Chain"),

("Back_Gears", "Back_Wheel"),

("Back_Wheel", "Frame")

**The problem selected for troubleshooting:** "The Back_Wheel is not spinning."

**Potential ground truth error locations that could have caused this problem:**

Malfunctioning connections between the following parts**:**

- ("Pedals", "Crank_Arm"),
- ("Crank_Arm", "Front_Gears"),
- ("Front_Gears", "Chain"),
- ("Back_Gears", "Chain"),
- ("Back_Gears", "Back_Wheel"),
- ("Frame", "Back_Gears"),
- ("Back_Wheel", "Frame"),
- ("Front_Gears", "Frame"),

Malfunctioning parts:

- Pedals
- Crank_Arm,
- Front_Gears
- Chain
- Back_Gears
- Back_Wheel
- Frame

.

**Figure 6**


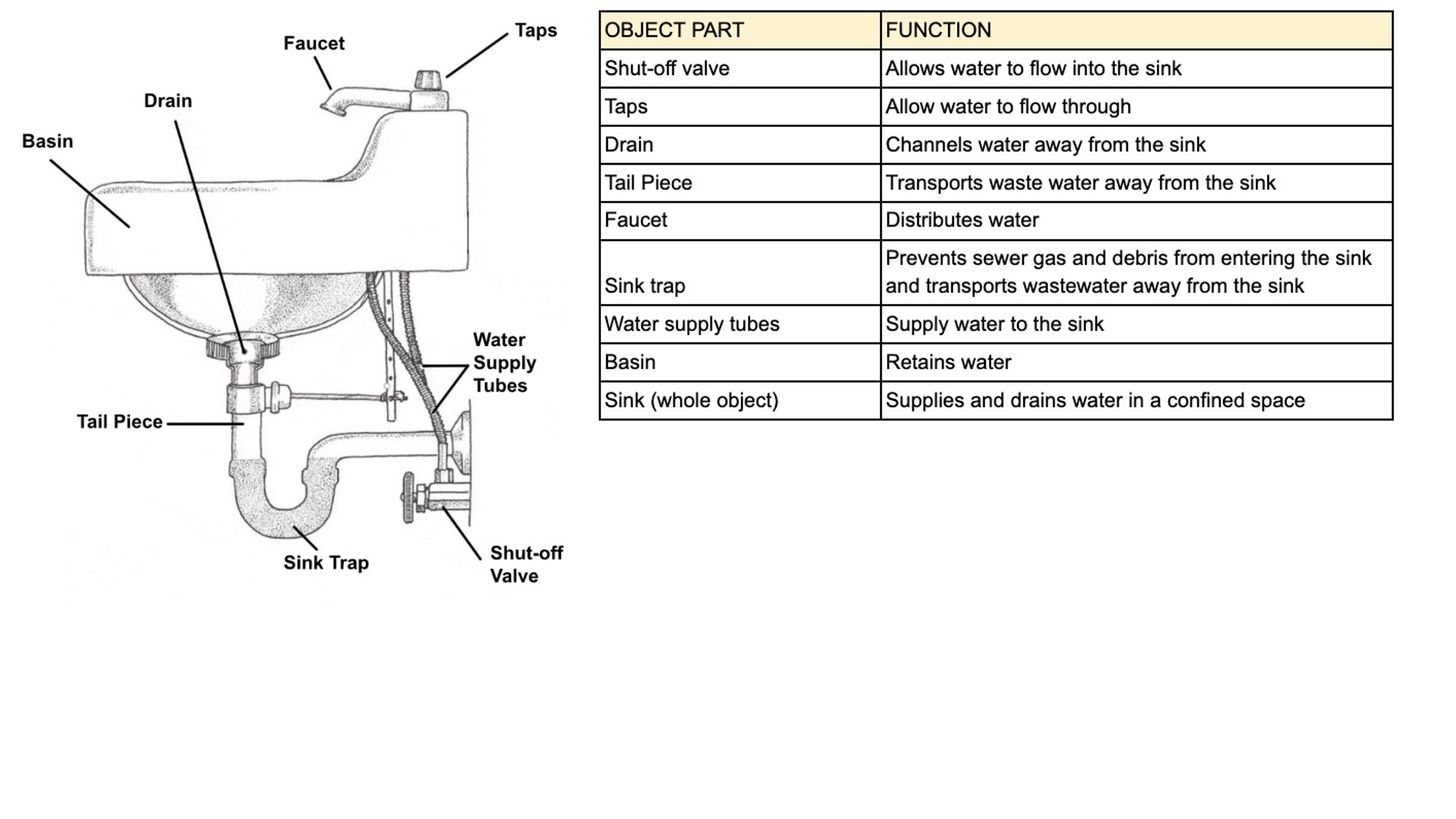
The diagram, part/function table, and the ground truth data for the sink.

**Ground truth causal model:**


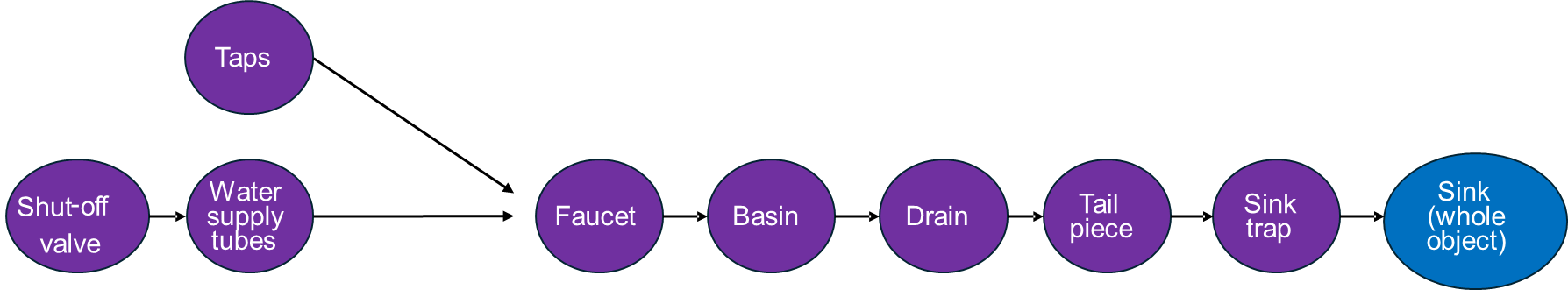


**Ground truth assembly plan as part connections:**

("Shut_off_valve", "Water_supply_tubes"),

("Water_supply_tubes", "Faucet"),

("Taps", "Faucet"),

("Sink_trap", "Tail_Piece"),

("Tail_Piece", "Drain"),

("Drain", "Basin")

**The problem selected for troubleshooting:** "No water is coming out"

**Potential ground truth error locations that could have caused this problem:**

Malfunctioning connections between the following parts**:**

- ("Taps", "Faucet"),
- ("Water_supply_tubes", "Faucet"),
- ("Shut_off_valve", "Water_supply_tubes"),

Malfunctioning parts:

- Taps
- Faucet
- Water_supply_tubes"
- Shut_off_valve

**Figure 7**


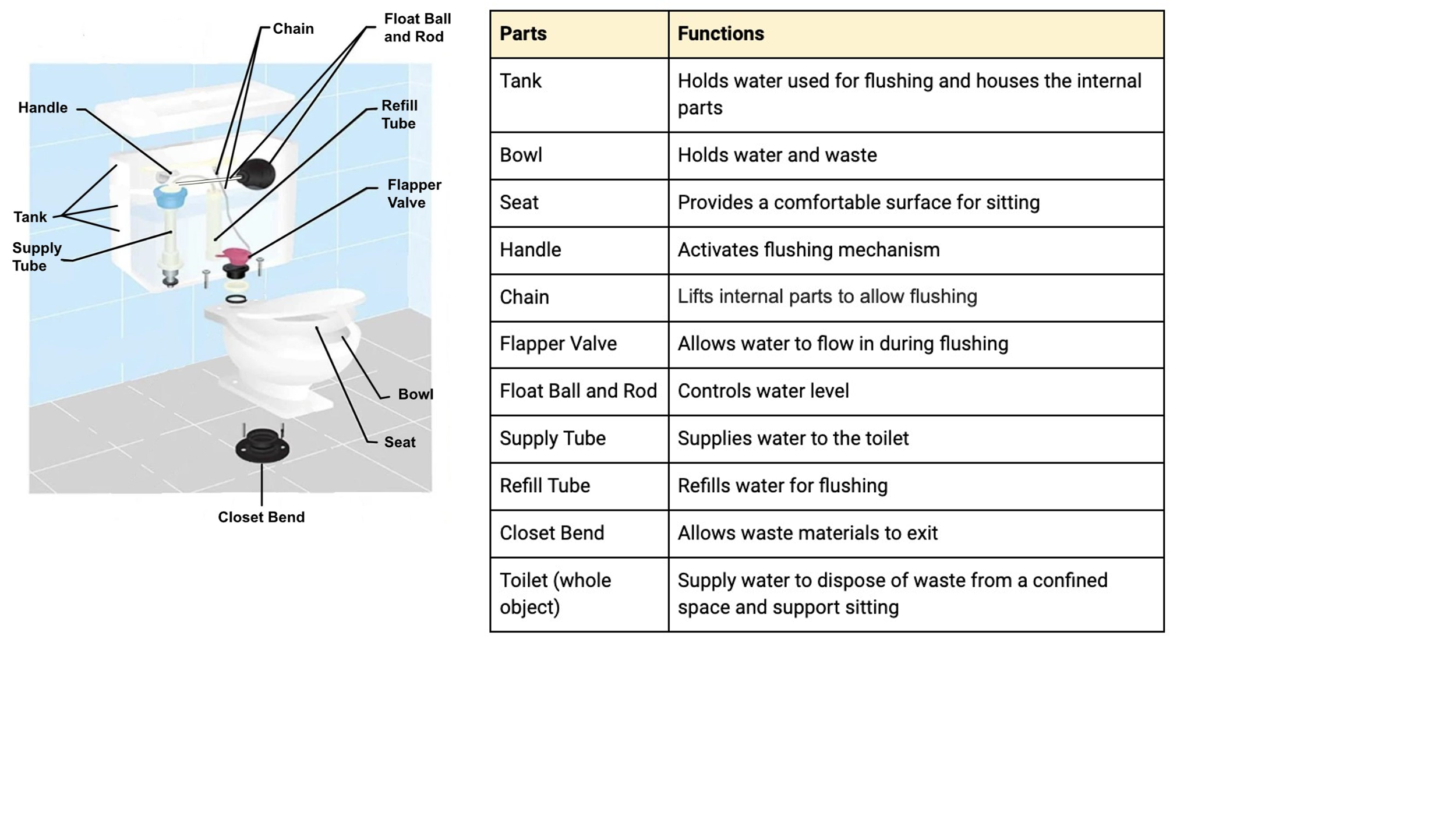
The diagram, part/function table, and the ground truth data for the toilet.

**Ground truth causal model:**


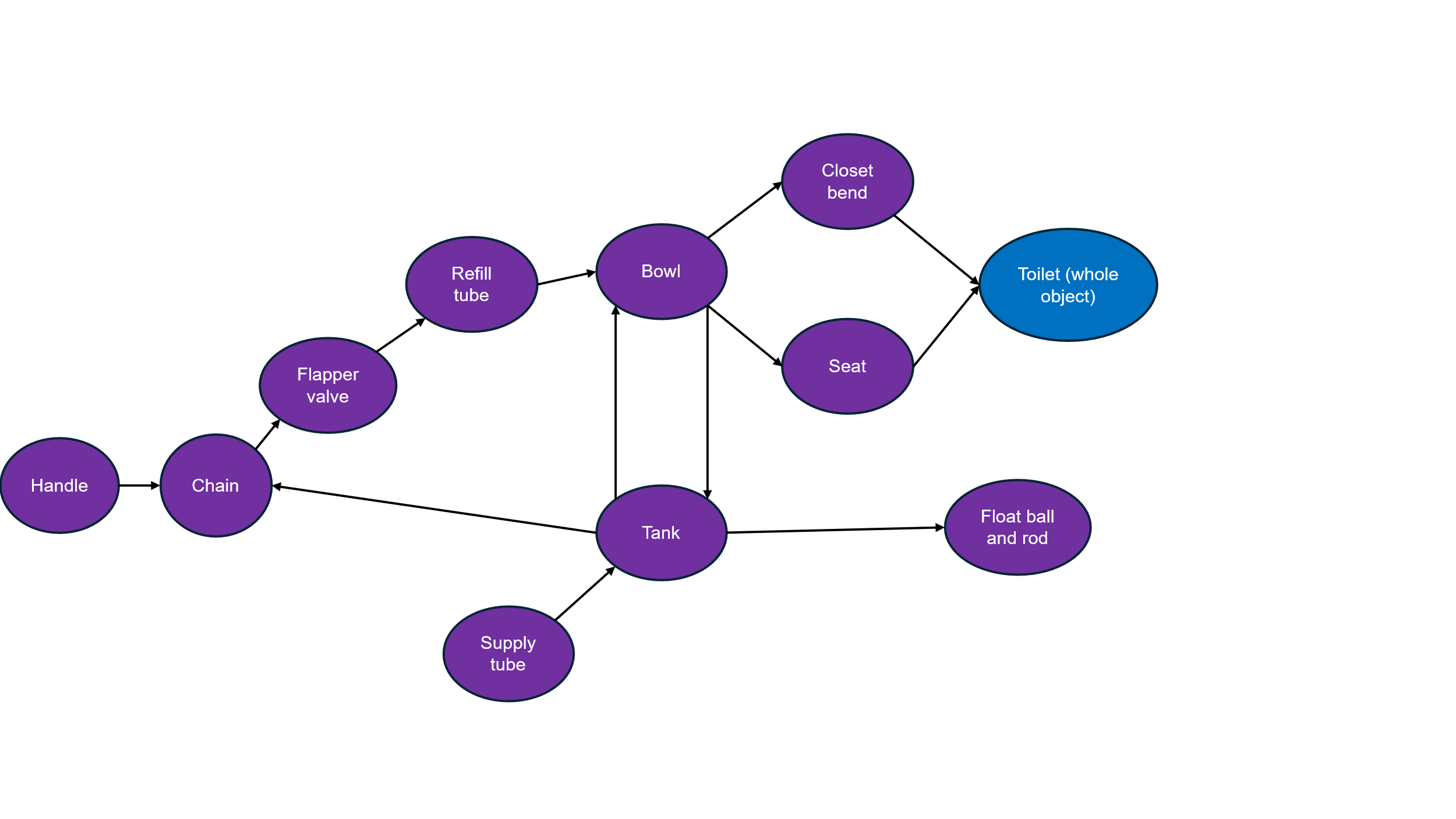


**Ground truth assembly plan as part connections:**

("Tank", "Supply_Tube"),

("Supply_Tube", "Float_Ball_and_Rod"),

("Tank", "Handle"),

("Handle", "Chain"),

("Chain", "Flapper_Valve"),

("Refill_Tube", "Flapper_Valve"),

("Flapper_Valve", "Bowl"),

("Bowl", "Seat"),

("Bowl", "Closet_Bend"),

("Refill_Tube", "Bowl"),

("Flapper_Valve", "Tank")

**The problem selected for troubleshooting:** "No water flows in during flushing"

**Potential ground truth error locations that could have caused this problem:**

Malfunctioning connections between the following parts**:**

- ("Tank", "Handle"),
- ("Supply_Tube", "Tank"),
- ("Tank", "Flapper_Valve"),
- ("Handle", "Chain"),
- ("Flapper_Valve", "Chain"),
- ("Float_Ball_and_Rod", "Supply_Tube"),

Malfunctioning parts:

- Tank
- Handle
- Supply_Tube
- Chain
- Flapper_Valve
- Float_Ball_and_Rod
